# Blood Pressure Level and Risk of All-Cause Mortality in Patients With Kidney Failure on Maintenance Peritoneal Dialysis: A Systematic Review and Meta-Analysis of Observational Studies

**DOI:** 10.1016/j.xkme.2025.101193

**Published:** 2025-11-26

**Authors:** Viangkaeo Lee, Haleh Siami, Vanessa-Giselle Peschard, Mohsen Malekinejad, Emily Huang, Peggy Tahir, Chi-yuan Hsu

**Affiliations:** 1 University of California, San Francisco, CA; 2 Islamic Azad University of Medical Sciences, Tehran, Iran; 3 Bates College, Lewiston, ME

**Keywords:** All-cause mortality, blood pressure, hypertension, kidney failure, peritoneal dialysis

## Abstract

**Rationale & Objective:**

To identify, appraise, and synthesize relevant epidemiologic studies to better understand the relations between blood pressure (BP) and outcomes in patients with kidney failure receiving maintenance peritoneal dialysis (PD) and to examine how the available evidence aligns with current clinical guidelines.

**Study Design:**

Systematic review and meta-analysis.

**Setting & Participants:**

We conducted a comprehensive search of 5 databases (PubMed, Web of Science, Embase, CINAHL, Cochrane Library) through April 2023 (and abstracts from leading nephrology conferences from 2019-2023). Studies were eligible if they included patients receiving maintenance PD and reported associations between BP levels and all-cause mortality.

**Exposures:**

Systolic BP, diastolic BP, or presence/absence of hypertension.

**Outcome:**

All-cause mortality.

**Analytical Approach:**

Dual independent screening and full-text review were performed. Where appropriate, data were then pooled using a random-effects meta-analysis. Adjusted data were not required for inclusion.

**Results:**

Thirty observational studies were included in the systematic review. Twenty-three studies were retrospective; 7 were prospective. Sixteen studies were single center, 13 were multicenter, and one study was unclear as it was presented only in abstract form. More than two-thirds were from Asia. Of the 28 full-length articles, only 9 had BP as the primary exposure, with the rest including BP only as a covariate in multivariable models. In the meta-analysis, compared with systolic BP 100-140 mm Hg, systolic BP >140 mm Hg was associated with a statistically nonsignificant 15% higher risk of all-cause mortality (RR, 1.15; 95% CI, 0.99-1.34). No association was found between diastolic BP >90 mm Hg (vs diastolic BP ≤90 mm Hg) and mortality (RR, 0.92; 95% CI, 0.53-1.57).

**Limitations:**

High level of heterogeneity and high risk of bias among the included studies.

**Conclusions:**

The existing epidemiology literature is unable to offer definitive guidance regarding BP treatment targets for PD patients due to heterogeneity, risk of bias, and lack of statistical significance.

## Plain-language Summary

Some individual studies have reported that among patients with kidney failure treated with chronic peritoneal dialysis, higher blood pressure level is not correlated with higher risk of death, which differs from what is observed in the general population. However, experts continue to recommend lowering blood pressure as a treatment. We systematically reviewed and summarized the medical literature published before April 2023 concerning blood pressure level and risk of death among patients with kidney failure treated with chronic peritoneal dialysis. We found that the published studies have numerous limitations, and we concluded that more research is needed to generate better data to help physicians decide how to best manage blood pressure in this vulnerable high-risk population.

Patients with kidney failure receiving maintenance peritoneal dialysis (PD) experience high rates of mortality.^1^^,^^2^ In the general population, stepwise increases in systolic blood pressure (SBP) levels are associated with gradually increasing risk of death, and randomized controlled trials have demonstrated that lowering SBP reduces the rates of all-cause mortality.^3^, ^4^, ^5^, ^6^, ^7^, ^8^

However, for patients receiving maintenance PD, multiple epidemiologic studies have reported that the relationship between SBP level and mortality is U-shaped, with elevated risk at lower SBP levels. Some nephrology opinion leaders have argued that since patients with kidney failure receiving dialysis face high short-term mortality due to more acute problems such as undernutrition and inflammation, they may not live long enough to experience the consequences of hypertension, and aggressive antihypertensive therapy may not be necessary.^9^

The International Society for Peritoneal Dialysis guidelines currently recommend that patients with blood pressure (BP) consistently >140/90 mm Hg be treated to maintain SBP <140 mm Hg and diastolic BP (DBP) <90 mm Hg (1D recommendation), although this recommendation is based on ‘very low’ quality evidence.^10^, ^11^, ^12^, ^13^ As there are no clinical trials, we conducted this systematic review and meta-analysis to identify, appraise, and synthesize relevant epidemiologic studies to assess how the current evidence aligns with existing clinical guidelines.

## Methods

We registered our protocol in the PROSPERO database (CRD42023416761)^14^^,^^15^ and followed Preferred Reporting Items for Systematic Reviews and Meta-Analyses^16^ guidelines for reporting. This study analyzed deidentified information from existing research and so was considered exempt human subjects research, and informed consent was not required.

### Eligibility Criteria for Systematic Review

#### Population

Eligible studies included patients of all age groups with kidney failure undergoing maintenance PD, irrespective of the underlying causes of kidney failure or the specific subtype of PD (eg, continuous ambulatory or automated). We excluded studies that combined patients receiving both hemodialysis and PD that did not report outcomes and associations stratified by modality.

#### Exposure

The primary exposure of interest was BP level, including systolic, diastolic, and mean arterial pressure.

#### Comparator

Comparators consisted of different levels of BP (discussed later) and were categorized into continuous, ordinal, or dichotomous variables (eg, presence vs absence of hypertension).

#### Outcome

Our outcome of interest was all-cause mortality. We excluded studies in which all-cause mortality was captured only as part of a composite outcome or studies that solely reported subtypes of mortality (eg, only deaths from cardiovascular disease).

#### Search Strategy and Study Selection

We collaborated with a research librarian (PT) to develop search strategies (Tables S1 and S2) in April 2023. We searched 5 databases: PubMed, Web of Science, Embase, CINAHL, and the Cochrane Library. The main concepts included PD, hypertension, and outcomes related to adverse events, mortality, or survival. We also reviewed the references of selected articles to ensure no important studies were missed.

After duplicate removal, 2 authors (VL and EH) independently reviewed titles, abstracts, and descriptor terms. Potentially eligible studies proceeded to the next review phase, when 2 different authors (CH and HS) independently examined the full text of each article. Any discrepancies were resolved through discussion.

We also conducted searches of conference abstracts from the American Society of Nephrology, International Society of Nephrology, and National Kidney Foundation meetings held from 2019 to 2023.

### Data Extraction

Among the studies that met our inclusion criteria, 2 authors (VL and EH) independently extracted data into a prestructured data extraction form (Table 1^11^, ^12^, ^13^^,^^17^, ^18^, ^19^, ^20^, ^21^, ^22^, ^23^, ^24^, ^25^, ^26^, ^27^, ^28^, ^29^, ^30^, ^31^, ^32^, ^33^, ^34^, ^35^, ^36^, ^37^, ^38^, ^39^, ^40^, ^41^, ^42^, ^43^). If there were any missing data, we attempted to contact the study authors. Third and fourth reviewers (CH and HS) then cross-verified the data extracted, corrected errors, and reconciled any disagreements as they arose.

**Table 1:** Characteristics of Studies Evaluating the Association Between Blood Pressure and All-Cause Mortality Included in the Systematic Review

Study	Study Location (Setting)	Data Collection Years	Demographics	Primary Cause of ESKD	BP Distribution (BP Measurement Method/Definition)	PD Status (Subtype)	Study design (N)	Deaths (n)	Follow-Up Period	Type of BP	Control Condition (mm Hg)	Risk Factor (mmHg)	BP Variable Type^a^	Effect Size^b^ (95% CI)	Confounders Adjusted For
**Peer-reviewed studies (n = 28)**															
Afshinnia^17^ (2016)	United States (Single center)	2007-2012	Mean age: 51.00 ± 15.00 y Pediatric patients: 0% Female: 49.40% Race/ethnicity: 77.9% White, 19.5% Black, 2.6% Other	DM: 31% HTN: 5% Glomerulopathy: 31% Polycystic kidney disease: 7% Genitourinary reflux: 5% Other: 21%	14% SBP <120 mm Hg 31% SBP 120-139 mm Hg 35% SBP 140-159 mm Hg 19% SBP ≥160 mm Hg (BP measurement method/definition not reported)	Incident PD patients (48.05% CAPD, 51.95% APD)	Retrospective cohort (77)	20	Median 35 mo (IQR, 21-58)	SBP	140-159	<120	Ordinal, static	HR, 0.620 (0.125-3.079)	Not adjusted
													Ordinal, time-varying	HR, 3.445 (0.671-17.695)	Adjusted for albumin and low ejection fraction
												120-139	Ordinal, static	HR, 1.313 (0.455-3.788)	Not adjusted
													Ordinal, time-varying	HR, 1.545 (0.302-7.901)	Adjusted as above
												≥160	Ordinal, static	HR, 1.706 (0.477-6.103)	Not adjusted
													Ordinal, time-varying	HR, 1.264 (0.162-9.883)	Adjusted as above
										DBP	80-89	<70	Ordinal, static	HR, 1.645 (0.456-5.934)	Not adjusted
													Ordinal, time-varying	HR, 4.224 (0.909-19.625)	Adjusted as above
												70-79	Ordinal, static	HR, 1.277 (0.427-3.816)	Not adjusted
													Ordinal, time-varying	HR, 1.840 (0.300-11.289)	Adjusted as above
												>90	Ordinal, static	HR, 0.634 (0.158-2.548)	Not adjusted
													Ordinal, time-varying	HR, 0.122 (0.040-0.372)	Adjusted as above
Akhlaghi^18^ (2013)	Iran (Multicenter)	1996-2009	Mean age: 50 ± 17 y Pediatric patients: 0% Female: 55.00% Race/ethnicity: NR	DM: 33.6% HTN: 35.4% Glomerulonephritis: 7.1% Polycystic kidney disease: 3.8% Vascular collagen disease: 0.5% Other: 19.6%	NR (BP measurement method/definition NR)	Prevalent PD patients (100% CAPD)	Retrospective cohort (2,006)	507	≤131 mo	SBP	<150	≥150	Dichotomous, static	HR^c^, 0.92 (0.73-1.17)	Adjusted for sex, age, BMI, DM, medical center, cholesterol, LDL, HDL, PTH, nPCR, TG, albumin, hemoglobin, creatinine, calcium, phosphorus, and SBP/DBP
										DBP	<100	≥100	Dichotomous, static	HR, 1.03 (0.70-1.51)	
Ateş^19^ (2001)	Turkey (Single center)	1992-1996	Median age: 46.3 ± 14.8 y	DM: 10.4% Hypertensive nephrosclerosis: 5.6% Glomerulonephritis: 27.2% Autosomal dominant polycystic kidney disease: 6.4% Chronic tubulointerstitial nephritis: 29.6% Renal amyloidosis: 8.8% Unknown: 12%	SBP (mm Hg): 131.3 ± 17.5 (HTN was defined as SBP >140 mm Hg or DBP >90 mm Hg or taking ≥2 drugs to control BP)	Incident PD patients (93% CAPD, 7% APD)	Retrospective cohort (125)	22	Mean 30.6 mo (range 7-40)	SBP	Not applicable	Per 10 mmHg increase	Continuous, time-averaged	RR, 1.64 (1.22-2.21)	Not adjusted
										DBP	Not applicable	Per 10 mm Hg increase	Continuous, time-averaged	RR 1.70 (1.,10-2.61)	Not adjusted
										Presence vs. absence of HTN	Absence of HTN	Presence of HTN	Dichotomous, time-averaged	RR, 6.01 (2.21-16.32)	Not adjusted
														RR, 5.61 (1.97-15.93)	Adjusted for age, total sodium removal, DM, comorbidity score, serum creatinine and albumin, nPNA, hematocrit, peritonitis rate, high transporter status, RRF, Kt/V urea, TCC, total sodium removal, total fluid removal, and hypertensive status
Ateş^20^ (2005)	Turkey (Single center)	NR	Median age: 42.1 ± 14.1 y (range 18-74) Pediatric patients: 0% Female: 45.36% Race/ethnicity: NR	DM: 17.52% Hypertensive nephrosclerosis: 9.28% Glomerulonephritis: 30.9% Autosomal dominant polycystic kidney disease: 4.12% Chronic tubulointerstitial nephritis: 25.77% Unknown: 12.37%	MAP (mm Hg): 99.7 ± 14.9 (SBP and DPB measured in the supine position after 10 min rest during routine visits, every 2 mo)	Incident PD patients (84% CAPD, 16% APD)	Retrospective cohort (97)	18	≤33.9 mo	MAP	Not applicable	Per 10 mm Hg increase	Continuous, static	RR, 0.86 (0.65-1.14)	Not adjusted
													Continuous, time-averaged	RR, 0.82 (0.58-1.16)	
Bao^21^ (2019)	China (Single center)	2005-2018	Mean age: 57.55 ± 13.08 y Pediatric patients: 0% Female: 48% Race/ethnicity: 100% Asian	DM: 36% HTN: 8% Glomerulonephritis: 29% Autosomal dominant polycystic kidney disease: 3% Chronic interstitial nephritis: 12% Unknown/other: 12%	SBP (mm Hg): 146 DBP (mm Hg): 84 (Brachial BP was measured twice in sitting position after patients had rested >10 min. SBP and DBP were average of 2 measurements)	Incident PD patients (subtype NR)	Retrospective cohort (181)	102	Median 46 mo (IQR 28-90)	SBP	Not applicable	Per 1 mm Hg increase	Continuous, static	HR, 1.00 (1.99-1.01)	Not adjusted
										DBP	Not applicable	Per 1 mm Hg increase	Continuous, static	HR, 0.98 (0.96-0.99)	Not adjusted
Beduschi^22^ (2015)	Brazil (Multicenter)	2004-2011	Mean age: 59.15 ± 16 y Pediatric patients: 0% Female: 45.50% Race/ethnicity: 50% White	DM: 36.75% HTN: 17.85% Glomerulonephritis: 9.45% Other: 18.1% Unknown: 17.85%	NR (HTN defined according to the WHO/ISH criteria, SBP >140 mm Hg and/or DBP >90 mm Hg at baseline with or without use of hypertensive medication)	Incident PD patients (50% CAPD, 50% APD)	Retrospective cohort (2,890)	550	≤74 mo	Presence vs. absence of HTN	Absence of HTN	Presence of HTN	Dichotomous, static	HR, 0.98 (0.80-1.20)	Adjusted for age, year of entry in PD, BMI, center experience, DM, literacy, sex, race, and length of predialysis care
														Sub-distribution HR^d^, 0.99 (0.81-1.22)	Adjusted for competing risks: (1) mortality, any cause of dropout from therapy except death; (2) technique failure, any cause of dropout from therapy except switching from PD to HD and (3) for time to first peritonitis any cause of dropout occurred before the first episode of peritonitis
Cao^23^ (2015)	China (Multicenter)	2012	Mean age: 52.2 ± 15.2 y Pediatric patients: 0% Female: 60.20% Race/ethnicity: NR	NR	SBP (mm Hg): 145.1 ± 20.1 DBP (mm Hg): 86.4 ± 12.9 (BP was measured at office within 3 mo before PD initiation, and ≥2 times/wk)	Incident PD patients (100% CAPD)	Retrospective cohort (5,405)	371	≤12 mo	DBP	Not applicable	Per 1 mm Hg increase	Continuous, static	HR, 0.387 (0.195-0.768)	Not adjusted
														HR, 0.426 (0.194-0.932)	Adjusted for sex, DM, RRF at the start of PD, DBP, Kt/V, PET type, and serum albumin level
Chaichaya^24^ (2020)	Thailand (Single center)	2011-2018	Mean age: 56.0 ± 12.7 y Pediatric patients: Included, but number not specified Female: 51.80% Race/ethnicity: NR	NR	SBP (mm Hg): 143.14 ± 22.62 DBP (mm Hg): 77.63 ± 12.45 (BP measurement method/definition NR)	Incident PD patients (subtype NR)	Retrospective cohort (828)	NR	NR	SBP	Not applicable	Per 1 mm Hg increase	Continuous, static	HR, 1.00 (0.99-1.01)	Adjusted for sex, age, DM, HTN, CVD, BMI, BP, albumin, bicarbonate, and hypokalemia
										DBP	Not applicable	Per 1 mm Hg increase	Continuous, static	HR, 0.99 (0.98-1.00)	
Chen^12^ (2022)	China (Single center)	2008-2018	Mean age: 54.3 ± 16.8 y Pediatric patients: 0% Female: 39.81% Race/ethnicity: NR	NR	SBP (mm Hg): 140 (IQR 130-155) DBP (mm Hg): 80 (IQR 73-90) (BP recorded at baseline and collected semiannually)	Incident PD patients (93.5% CAPD, 6.5% other)	Prospective cohort (216)	56	Median 65.9 mo (IQR, 31.9-87.4)	MAP	Not applicable	Per 1 mm Hg increase	Continuous, static	HR, 0.998 (0.967-1.030)	Not adjusted
Dai^25^ (2020)	China (Single center)	2001-2019	Median age: 63.61 y (IQR 46.64-73.2) Pediatric patients: 0% Female: 40.8% Race/ethnicity: NR	NR	NR (BP was assessed for 24 h. Daytime [7 AM-11 PM] BP measured every 10-20 min and nighttime [11 PM-7 AM] BP measured every 30-60 min. Ambulatory recordings had to contain >80% of valid readings for further analysis.)	Both incident and prevalent PD patients (subtype NR)	Prospective cohort (260)	64	Median 40.7 mo (IQR 26.1-67.8)	SBP	Not applicable	Per 1 mm Hg increase	Continuous, static	HR, 1.014 (1.002-1.026)	Adjusted for age, sex, BMI, duration of PD, HbA_1c_, HOMA- IR, and albumin
										DBP	Not applicable	Per 1 mm Hg increase	Continuous, static	HR, 0.980 (0.958-1.002)	Not adjusted
Fang^26^ (2009)	Canada (Multicenter)	2000-2006	Mean age: 59.4 ± 17.4 y Pediatric patients: 0% Female: 46.10% Race/ethnicity: 59.8% White, 8.8% Black, 27.1% Asian, 4.2% Other	DM: 28.8% Hypertensive nephrosclerosis: 13.4%, Glomerulonephritis or autoimmune disease: 27.8% Polycystic kidney disease: 7.5% Other: 22.5%	SBP (mm Hg): 134.1 ± 22.6 DBP (mm Hg): 77.3 ± 13.6 (BP was measured in the PD clinic with the patient in the supine position. SBP and DBP were collected serially every 3 mo)	Incident PD patients (38.9% CAPD, 61.1% APD)	Retrospective cohort (306)	74	Median 21.1 mo (range 0.6-83.5)	SBP	Not applicable	Per 1 mm Hg increase	Continuous, static	HR, 0.978 (0.959-0.998)	Adjusted for age, sex, DM, previous CVD, pulse pressure, albumin, CHF, SBPAdjusted as above
										DBP	Not applicable	Per 1 mm Hg increase	Continuous, static	HR, 0.979 (0.960-0.999)	
Goldfarb-Rumyantzev^27^ (2005)	United States (Multicenter)	1996-1999	Mean age: 57.2 ± 15.3 y Pediatric patients: 0% Female: 48.00% Race/ethnicity: 72.2% White, 18.7% Black, 5.3% Asian, 1.4% Native American, 2.3% Other, 0.1% Unknown	DM: 45% HTN: 22% Glomerulonephritis: 9% Other: 24%	SBP (mm Hg): 141.66 ± 20.33 DBP (mm Hg): 79.86 ± 11.95 (BP measurement method/definition NR)	Incident PD patients (66.5% CAPD, 29.8% APD, 3.7% combined subtypes)	Retrospective cohort (1,053)	NR	Mean 23 ± 14 mo	SBP	111-120	≤100	Ordinal, static	HR^c^, 2.71 (1.54-4.77)	Adjusted for baseline age, Asian race, primary cause of ESKD, HTN, SBP/DBP, serum bicarbonate, serum albumin, calcium × phosphate product, RRF, dialysis creatinine clearance, volume drained, and variables that are considered to be potential confounders but did not reach significant association with the outcome (African American and Native American race, primary cause of ESKD, glomerulonephritis, height, number of antihypertensive medications, history of left ventricular hypertrophy and coronary artery disease; hemoglobin, cholesterol and TG levels, D/P 4 h ratio and total dialysate volume).
												101-110	Ordinal, static	HR, 1.85 (1.06-3.21)	
												121-130	Ordinal, static	HR, 1.25 (0.83-1.88)	
												131-140	Ordinal, static	HR, 1.03 (0.68-1.54)	
												141-160	Ordinal, static	HR^c^, 0.94 (0.64-1.38)	
												161-180	Ordinal, static	HR^c^, 1.27 (0.81-1.98)	
												>180	Ordinal, static	HR^c^, 1.03 (0.54-1.95)	
										DBP	76-80	≤65	Ordinal, static	HR, 1.25 (0.85-1.80)	
												66-75	Ordinal, static	HR, 1.30 (0.96-1.75)	
												81-85	Ordinal, static	HR, 1.08 (0.77-1.52)	
												86-90	Ordinal, static	HR, 1.12 (0.77-1.62)	
												91-100	Ordinal, static	HR, 0.99 (0.66-1.50)	
												101-110	Ordinal, static	HR, 1.28 (0.68-2.40)	
												>110	Ordinal, static	HR, 2.51 (0.86-7.38)	
Iliescu^28^ (2002)	Canada (Single center)	1999-2001	Mean age: 58.1 ± 15.9 y Pediatric patients: 0% Female: 55.6% Race/ethnicity: 88.88% White, 3.70% Hispanic, 5.55% Other	DM: 50% Nephrosclerosis: 11.11% Glomerulonephritis: 22.22% Polycystic kidney disease: 3.70% Interstitial nephritis: 3.70% Unknown: 9.25%	DBP (mm Hg): 79.6 ± 13.3 SBP (mm Hg): 138.2 ± 26.0 (BP measured in the sitting position with a mercury sphygmomanometer with an appropriately sized cuff)	Prevalent PD patients (28% CAPD, 72% APD)	Prospective cohort (54)	24	≤24 mo	SBP	Not applicable	Per 1 mm Hg increase	Continuous, static	RR, 0.99 (0.97-1.00)	Not adjusted
										DBP	Not applicable	Per 1 mm Hg increase	Continuous, static	RR, 0.95 (0.92-0.98)	Not adjusted
											≤70	≤70 = 0, >70 = 1	Dichotomous, static	RR, 0.34 (0.13-0.89)	Adjusted for age, DM, DBP, lipoprotein(a) level, serum albumin
Jager^29^ (1999)	Netherlands (Multicenter)	1993-1997	Mean age: 54 ± 14 y Pediatric patients: 0% Female: 36% Race/ethnicity: NR	DM: 16% HTN/renal vascular disease: 23%	SBP (mm Hg): 142 ± 22 DBP (mm Hg): 85 ± 11 MAP (mm Hg): 104 ± 13 (SBP, DBP, and heart rate were measured once at a routine visit in the outpatient clinic)	Incident PD patients (95% CAPD, 5% other)	Prospective cohort (118)	33	Mean 25 mo (range 4-44)	SBP	Not applicable	Per 10 mm Hg increase	Continuous, static	RR, 1.25 (1.09-1.45)	Not adjusted
														RR, 1.42 (1.17-1.73)	Adjusted for age and total creatinine appearance
										DBP	Not applicable	Per 10 mm Hg increase	Continuous, static	RR, 0.89 (0.66-1.21)	Not adjusted
										MAP	Not applicable	Per 10 mm Hg increase	Continuous, static	RR, 1.18 (0.92-1.51)	Not adjusted
Jhee^30^ (2018)	South Korea (Multicenter)	2009-2014	Mean age: 62.3 ± 13.0 y Pediatric patients: 0% Female: 43.20% Race/ethnicity: NR	NR	SBP (mm Hg): 133 ± 21 DBP (mm Hg): 80 ± 12 (BP measurement method/definition NR)	Prevalent PD patients (subtype NR)	Prospective cohort (924)	188	Median 56 mo (IQR 38-64)	SBP	130-139	<110	Ordinal, static	HR^c^, 1.45 (0.88-2.38)	Adjusted for age, sex, comorbidities, dialysis vintage and type, numbers of antihypertensive agents, serum albumin, and LDL-C
												110-119	Ordinal, static	HR, 0.61 (0.33-1.12)	
												120-129	Ordinal, static	HR, 0.81 (0.50-1.30)	
												140-149	Ordinal, static	HR^c^, 0.95 (0.60-1.52)	
												150-159	Ordinal, static	HR^c^, 0.50 (0.25-0.98)	
												160-169	Ordinal, static	HR^c^, 0.76 (0.32-1.79)	
												≥170	Ordinal, static	HR^c^, 1.38 (0.74-2.54)	
Kemperman^31^ (1991)	Netherlands (Multicenter)	1979-1989	Mean age: 47 y (range 21-72) Pediatric patients: 0% Female: 46% Race/ethnicity: 84% White, 16% Other	DM: 100%	SBP (mm Hg): 164 ± 22 DBP (mm Hg): 89 ± 12 (BP measurement method/definition NR)	Incident PD patients (100% CAPD)	Retrospective cohort (65)	32	NR	SBP	<160	≥160	Dichotomous, static	RR, 2.60 (CI NR)	Adjusted for age >45 y, previous or current cardiac disease, SBP ≥160 mmHg, type and duration of DM, DBP, and RRF
Liao^32^ (2011)	Taiwan (Single center)	1999-2009	Mean age: 52.8 ± 15.8 y Pediatric patients: 0% Female: 55.7% Race/ethnicity: 100% Asian	DM: 20% Hypertensive nephrosclerosis: 10% Glomerulonephritis: 41%	SBP (mm Hg): 143.0 ± 21.5 DBP (mm Hg): 83.5 ± 13.6 (HTN defined as BP ≥130/80 mm Hg)	Prevalent PD patients (subtype NR)	Retrospective cohort (280)	77	≤49.2 mo	Presence vs. absence of HTN	Absence of HTN	Presence of HTN	Dichotomous, static	HR^c^, 0.762 (0.364-1.598)	Not adjusted
Liu^33^ (2008)	Taiwan (Single center)	2005-2007	Mean age: 54.5 ± 14.2 y Pediatric patients: 0% Female: 68% Race/ethnicity: NR	DM: 20% Hypertensive nephrosclerosis: 10% Glomerulonephritis: 41%	SBP (mm Hg): 148.66 ± 18.55 DBP (mm Hg): 88.66 ± 14.66 (BP was measured from the arm after 20 min rest in the supine position. Two readings were taken 10 min apart, and SBP and DBP were recorded as the average of the 2 measurements. BP was measured during 3 sequential clinical visits, per month)	Prevalent PD patients (subtype NR)	Prospective cohort (153)	27	≤33 mo	SBP	Not applicable	Per 1 mm Hg increase	Continuous, static	HR, 1.12 (1.02-1.22)	Adjusted for sex, age, and RRF status
										DBP	Not applicable	Per 1 mm Hg increase	Continuous, static	HR, 0.92 (0.85-0.98)	
Lyu^34^ (2019)	China (Single center)	2008-2016	Mean age: 49 ± 15 y Pediatric patients: 0% Female: 43.30% Race/ethnicity: NR	DM: 13% Hypertensive nephrosclerosis: 5% Glomerulonephritis: 63% Polycystic kidney disease: 2% Obstructive uropathy: 2% Other: 16%	MAP (mm Hg): 105 ± 12 (During each outpatient follow-up, BP was measured with an electronic cuff sphygmomanom eter, and MAP was calculated from the measurements)	Incident PD patients (subtype NR)	Retrospective cohort (1,737)	208	Median 33 mo (IQR 19.3-52.4)	MAP	96-119	≤95	Ordinal, static	HR, 1.40 (1.01-1.93)	Adjusted for age, sex, comorbidities, BMI, primary cause of kidney disease, hemoglobin level, albumin level, RRF, antihypertensive medications, and diuretics
												≥120	Ordinal, static	HR, 2.12 (1.32-3.40)	
Park^35^ (2010)	South Korea (Single center)	2004-2008	Mean age: 51.6 ± 9.9 y Pediatric patients: 0% Female: 53.77% Race/ethnicity: NR	HTN: 22.6% Glomerulonephritis: 25.5% Other: 12.3% Unknown: 39.6%	SBP (mm Hg): 141.94 ± 16.04 DBP (mm Hg): 84.41 ± 9.67 (HTN was defined as SBP ≥130 mm Hg and/or DBP ≥85 mm Hg)	Prevalent PD patients (100% CAPD)	Prospective cohort (106)	15	≤60 mo	Presence vs. absence of HTN	Absence of HTN	Presence of HTN	Dichotomous, static	RR, 7.04 (0.79-62.59)	Adjusted for age, sex, albumin, and hematocrit
Prasad^36^ (2013)	India (Single center)	2004-2011	Mean age: 45.1 ± 16.2 y Pediatric patients: 0% Female: 36.20% Race/ethnicity: 100% Asian	NR	SBP (mm Hg): 134.5 ± 14.9 DBP (mm Hg): 86.5 ± 8.9 (HTN was defined as SBP ≥130 mm Hg and/or DBP ≥85 mm Hg)	Incident PD patients (subtype NR)	Retrospective cohort (163)	32	Mean 24 ± 14 mo (range 6-65)	SBP	<130	≥130	Dichotomous, static	HR, 2.65 (1.22-6.27)	Not adjusted
														HR^c^, 1.60 (0.38-6.70)	Adjusted for age, sex, comorbidities, serum albumin, peritonitis, Subjective Global Assessment score
										DBP	<85	≥85	Dichotomous, static	HR, 3.12 (1.46-6.68)	Not adjusted
													Dichotomous, static	HR^c^,1.48 (0.41-5.28)	Adjusted as above
										Presence vs. absence of HTN	Absence of HTN	Presence of HTN	Dichotomous, static	HR, 4.22 (1.87-9.54)	Not adjusted
													Dichotomous, static	HR, 2.23 (0.36-13.83)	Adjusted as above
Qiu^37^ (2020)	China (Single center)	2006-2013	Mean age: 47.5 ± 15.3 y Pediatric patients: 0% Female: 40.20% Race/ethnicity: NR	DM: 22.8% HTN: 7.2% Glomerulonephritis: 61.1% Other: 8.9%	SBP (mm Hg): 137 ± 20 DBP (mm Hg): 85 ± 14 (BP measurement method/definition NR)	Incident PD patients (100% CAPD)	Retrospective cohort (1,656)	507	Median 46.5 mo (IQR, 2.6-154.3)	SBP	110-130	<110	Ordinal, static	HR^c^,1.51 (1.12-2.03)	Adjusted for age, sex, history of CVD, diabetic status, hemoglobin, serum albumin, 24-h urine volume, and antihypertensive medications
												>130	Dichotomous, static	HR^c^, 1.40 (1.11-1.76)	
											Not applicable	Per 5 mm Hg increase	Continuous, static	HR, 1.01 (0.99-1.03)	
										DBP	Not applicable	Per 5 mm Hg increase	Continuous, static	HR, 1.02 (0.98-1.05)	
										MAP	Not applicable	Per 5 mm Hg increase	Continuous, static	HR, 1.02 (0.99-1.05)	
Rocco^38^ (2002)	United States (Multicenter)	1996-1998	Mean age: 54.6 ± 14.9 y Pediatric patients: 0% Female: 49.00% Race/ethnicity: 65% White, 24% Black, 10% Hispanic	DM: 35% HTN: 23% Glomerulonephritis: 17%	NR (BP measurement method/definition NR)	Prevalent PD patients (53% CAPD, 34% cycler)	Retrospective cohort (1,187)	200	≤12 mo	SBP	>151	80-123	Ordinal, static	OR, 1.14 (0.73-1.77)	Not adjusted
												124-137	Ordinal, static	OR^c^, 0.92 (0.58-1.45)	
												138-151	Ordinal, static	OR, 0.75 (0.46-1.21)	
										DBP	>88	40-72	Ordinal, static	OR, 3.12 (1.91-5.10)	Not adjusted
													Ordinal, static	HR^c^, 1.86 (1.19-2.92)	Adjusted for age, race(Black vs White only), Hispanic ethnicity, DM as the primary cause of ESKD (vs all other causes combined), duration of dialysis, quartile mean serum albumin (BCG laboratory method only), mean D/P creatinine from PET data, quartile mean body surface area, quartile mean DBP, quartile mean SBP, modality status (CAPD vs cycler), and mean hematocrit
												73-80	Ordinal, static	OR 1.95 (1.1,7-3.25)	Not adjusted
													Ordinal, static	HR^c^, 1.48 (0.92-2.36)	Adjusted as above
												81-88	Ordinal, static	OR, 1.65 (0.99-2.76)	Not adjusted
													Ordinal, static	HR, 1.29 (0.81-2.06)	Adjusted as above
Udayaraj^39^ (2009)	United Kingdom (Multicenter)	1997-2006	Median age: 58.0 y (IQR, 44.6-68.6) Pediatric patients: 0% Female: 42% Race/ethnicity: 89.4% White, 2.8% Black, 6.8% Asian, 1.2% Other	DM: 19.8% HTN: 6.1% Glomerulonephritis: 14.6% Polycystic kidney disease: 8.9% Pyelonephritis: 8.8% Renovascular disease: 5.4% Unknown: 23.4% Other: 13.0%	SBP (mm Hg): 143.1 ± 21.9 DBP (mm Hg): 81.4 ± 12.5 MAP (mm Hg): 101.9 ± 14.0 (Mean of BP values from the first 2 quarters of renal replacement therapy were used as the basis for this study)	Incident PD patients (subtype NR)	Retrospective cohort (2,770)	1,104	Median 44.4 mo (range 1.2-118.8)	SBP	Not applicable	Per 10 mm Hg increase	Continuous, static	HR, 0.88 (0.80-0.96)	Not adjusted
													Continuous, static	HR, 0.84 (0.78-0.92)	Adjusted for age; sex; ethnicity (White, Black, Asian, and other); primary renal disease causing ESKD; baseline (mean of the values from the first 2 quarters) hemoglobin, calcium, and phosphate levels; and time taken to be registered on the deceased-donor transplant waiting list from start of renal replacement therapy
										DBP	Not applicable	Per 10 mm Hg increase	Continuous, static	HR, 0.68 (0.59-0.79)	Not adjusted
													Continuous, static	HR, 0.78 (0.67-0.91)	Adjusted as above
										MAP	Not applicable	Per 10 mm Hg increase	Continuous, static	HR, 0.73 (0.64-0.84)	Not adjusted
													Continuous, static	HR, 0.77 (0.67-0.87)	Adjusted as above
Vejakama^40^ (2013)	Thailand (Multicenter)	2008-2011	Mean age: 50 ± 14 y Pediatric patients: 0% Female: 49.90% Race/ethnicity: NR	NR	SBP (mm Hg): 141 ± 25 DBP (mm Hg): 81 ± 14 (BP measurement method/definition NR)	Incident PD patients (100% CAPD)	Retrospective cohort (1,177)	188	Median 22.9 mo (range 1.8-43.5)	SBP	<140	≥140	Dichotomous, time-varying	HR, 0.83 (0.62-1.10)	Not adjusted
													Dichotomous, time-varying	HR, 0.70 (0.51-0.95)	Adjusted for age, BMI, serum albumin, hemoglobin, ultrafiltration volume, SBP, and diabetes
													Dichotomous, time-varying	HR, 0.69 (0.51-0.94)	Adjusted for age, serum albumin, hemoglobin, ultrafiltration volume, and SBP
										DBP	<90	≥90	Dichotomous, time- varying	HR, 0.92 (0.67-1.24)	Not adjusted
										Presence vs. absence of HTN	Absence of HTN	Presence of HTN	Dichotomous, time-varying	HR, 1.06 (0.75-1.49)	Not adjusted
Wu^11^ (2023)	China (Multicenter)	2005-2018	Median age: 49.0 y (IQR, 39.0-61.0) Pediatric patients (%): Included, but number not specified Female: 42.10% Race/ethnicity: NR	DM: 19.8% HTN: 6.1% Glomerulonephritis: 14.6% Polycystic kidney disease: 8.9% Pyelonephritis: 8.8% Renovascular disease: 5.4% Unknown: 23.4% Other: 13.0%	SBP (mm Hg): 139.8 ± 25.7 DBP (mm Hg): 83.5 ± 15.8 (HTN was defined as SBP >140 mm Hg or DBP >90 mm Hg or the use of antihypertensive medications)	Incident PD patients (100% CAPD)	Retrospective cohort (3,073)	571	Median 33.7 mo (IQR, 15.6-60.9)	Presence vs. absence of HTN	Absence of HTN	Presence of HTN	Dichotomous, static	HR, 1.83 (1.47-2.29)	Adjusted for age, sex, BMI, SBP, current smoking, current alcohol consumption, underlying causes of ESKD, DM, hyperlipidemia, medications, hemoglobin, serum albumin, serum uric acid, RRF, cholesterol, and high-sensitivity C-reactive proteinAdjusted as above
													Dichotomous, static	HR^d^, 1.57 (1.25-1.98)	
Xie^41^ (2020)	China (Multicenter)	2008-2018	Mean age: 52.3 ± 14.6 y Pediatric patients: 0% Female: 44.30% Race/ethnicity: 100% Asian	DM: 9.3% Glomerulonephritis: 56.5% Renal vascular disease: 3.4% Obstructive nephropathy: 0.9% Polycystic kidney disease: 1.4% Other: 28.5%	SBP (mm Hg): 140.0 ± 17.1 DBP (mm Hg): 85.0 ± 11.0 MAP (mm Hg): 103.4 ± 11.5 (BP averaged over the first 3 mo after the initiation of PD therapy)	Incident PD patients (subtype NR)	Retrospective cohort (7,335)	1,281	Median 35.8 mo (IQR, 22.3-54.1 mo)	SBP	119-141	<119	Ordinal, static	HR^c^, 1.36 (1.12-1.64)	Adjusted for age, BMI, sex, education level, CVD, DM, malignancy, hemoglobin, serum albumin, RRF, antihypertensive treatment, use of diuretics and erythropoietin
													Ordinal, static	HR^c^^,^^d^, 1.38 (1.12-1.69)	Adjusted as above. Transfer to HD and receive kidney transplantation were regarded as competing events
												>141	Ordinal, static	HR^c^, 1.22 (1.08-1.37)	Adjusted as above
													Ordinal, static	HR^c^^,^^d^, 1.20 (1.07-1.35)	Adjusted as above. Transfer to HD and receive kidney transplantation were regarded as competing events
										DBP	68-85	<67	Ordinal, static	HR, 1.39 (1.15-1.69)	Adjusted as above.
													Ordinal, static	HR^d^, 1.43 (1.17-1.75)	Adjusted as above. Transfer to HD and receive kidney transplantation were regarded as competing events
												>85	Ordinal, static	HR^c^, 1.16 (1.02-1.31)	Adjusted as above
													Ordinal, static	HR^c^^,^^d^, 1.11 (0.97-1.25)	Adjusted as above. Transfer to HD and receive kidney transplantation were regarded as competing events
										MAP	89-102	<88	Ordinal, static	HR, 1.31 (1.09-1.57)	Adjusted as above
													Ordinal, static	HR^d^, 1.35 (1.12-1.62)	Adjusted as above. Transfer to HD and receive kidney transplantation were regarded as competing events
												>102	Ordinal, static	HR, 1.21 (1.07-1.37)	Adjusted as above
													Ordinal, static	HR^d^, 1.19 (1.05-1.34)	Adjusted as above. Transfer to HD and receive kidney transplantation were regarded as competing events
Xu^13^ (2021)	China (Single center)	2006-2020	Mean age: 57.80 ± 15.13 y Pediatric patients: 0% Female: 42.29% Race/ethnicity: NR	NR	SBP (mm Hg): 148.41 ± 26.49 DBP (mm Hg): 81.99 ± 17.12 (BP measurement method/definition NR)	Incident PD patients (subtype NR)	Retrospective cohort (376)	70	≤168 mo	SBP	Not applicable	Per 1 mm Hg increase	Continuous, static	HR, 1.013 (1.005-1.021)	Not adjusted
													Continuous, static	HR, 1.007 (0.997-1.017)	Adjusted for patient demographics and important laboratory indicators
										DBP	Not applicable	Per 1 mm Hg increase	Continuous, static	HR, 0.99 (0.98-1.01)	Not adjusted
**Abstracts (n = 2)**															
Kim^42^ (2022)	South Korea (NR)	2000-2019	Mean age: NR Pediatric patients: 0% Sex (%F): NR Race/ethnicity: NR	NR	NR (BP measurement method/definition NR)	Incident PD patients (subtype NR)	Retrospective cohort (490)	102	NR	SBP	120-140	<120	Ordinal, static	HR^c^, 3.30 (1.70-6.40)	Adjusted for sex, DM, BMI, and previous coronary artery diseases
												140-160	Ordinal, static	HR^c^, 1.68 (0.83-3.40)	
												≥160	Ordinal, static	HR^c^, 2.30 (1.10-4.90)	
Kurahashi^43^ (2020)	Japan (Single center)	2010-2017	Mean age: NR Pediatric patients (%): NR Sex (%F): NR Race/ethnicity: NR	NR	NR (HTN was defined as SBP ≥140 mm Hg, DBP ≥90 mm Hg, or use of any antihypertensive drugs)	NR	Retrospective cohort (416)	NR	Mean 29.2 mo (SD NR)	SBP	110-139	<110	Ordinal, static	HR^c^, 2.08 (1.23-3.51)	Adjusted for age, sex, comorbidities, laboratory covariates, left ventricle ejection fraction. and medications such as renin-angiotensin system blockades and statins

*Note*: All studies reported relative effect sizes with 95% CIs, which were converted to the natural logarithmic scale for meta-analysis calculations. For presentation in tables and forest plots, these estimates were back-converted to the original scale, resulting in minor rounding discrepancies from the original study-reported CIs. Individual effect sizes for Afshinnia et al^17^ and Jhee et al^30^ were obtained through direct correspondence with the authors, as these data were not included in their original published studies. In the original data table of Prasad et al,^36^ the upper limit of the 95% CI for the effect size was incorrectly reported as being smaller than the point estimate (HR, 2.23; 95% CI, 0.359-1.869; *P* = 0.389). Using the reported point estimate, the lower limit of the 95% CI, and *P* value, the standard error was first back-calculated in natural logarithmic scale and then calculated and used the corrected upper limit of 13.829 in the meta-analysis for this study.

Abbreviations: APD, automated peritoneal dialysis; BCG, bromocresol green; BMI, body mass index; BP, blood pressure; CAPD, continuous ambulatory peritoneal dialysis; CHF, congestive heart failure; CVD, cardiovascular disease; D/P, dialysate-to-plasma ratio; DBP, diastolic blood pressure; DM, diabetes mellitus; ESKD, end-stage kidney disease; HbA_1c_, hemoglobin A_1c_; HD, hemodialysis; HDL, high-density lipoprotein; HOMA-IR, homeostatic model assessment of insulin resistance; HR, hazard ratio; HTN, hypertension; IQR, interquartile range; LDL, low-density lipoprotein; MAP, mean arterial pressure; nPCR, normalized protein catabolic rate; nPNA, normalized protein nitrogen appearance ; NR, not reported; OR, odds ratio; PD, peritoneal dialysis; PET, peritoneal equilibration test; PTH, parathyroid hormone; RR, risk ratio; RRF, residual renal function; SBP, systolic blood pressure; SD, standard deviation; TCC, total creatinine clearance; TG, triglycerides; WHO/ISH, World Health Organization/International Society of Hypertension.

a Static refers to BP ascertained at baseline (and not updated or averaged with later BP readings).

b Cox models unless otherwise specified.

c Included in the meta-analysis.

d Fine and Gray competing risk model.

### Bias Assessment

Two authors (CH and VP) independently assessed the final included studies using the risk of bias assessment tool for cohort studies developed by the Clinical Advances Through Research and Information Translation (CLARITY) group at McMaster University^44^ (Fig S1).

### Eligibility Criteria for Meta-Analysis

We performed several meta-analyses to investigate the association between BP measures and all-cause mortality based on 3 types of BP measures: (1) SBP only, (2) DBP only, and (3) the presence or absence of hypertension, which was defined using a combination of SBP or DBP or use of antihypertensive medications as defined by individual studies (Table 1). Adjusted data were not required for inclusion.

We did not include in the meta-analysis studies that assumed there was a linear relationship between BP level and adverse outcome (eg, studies that only reported change in risk per 10 mm Hg higher SBP). This is because several studies clearly showed that both lower and higher SBP levels were associated with higher risk of mortality,^37^^,^^41^ and this nonlinear relationship was borne out in the meta-analysis.

Most studies examined the association between BP level ascertained at baseline and the subsequent risk of all-cause mortality (labeled as ‘static’ under ‘BP variable type’ in Table 1). A few studies, however, treated BP as a time-varying exposure^17^^,^^40^ or averaged all BP readings prior to outcome.^19^^,^^20^ Due to concerns about bias introduced by the latter approaches (see Discussion), we limited our meta-analysis only to studies that examined the association between baseline BP level and subsequent risk of all-cause mortality.

#### Exposure: SBP

SBP was the main parameter of interest because it is the treatment target in contemporary clinical trials.^6^^,^^8^^,^^45^, ^46^, ^47^ We defined the SBP range of 100-140 mm Hg as the control condition, and patients with values higher than this range were considered the at-risk (exposure) group. To account for variability in how studies reported SBP ranges, we established a set of decision criteria. We considered any values falling between 100 and 140 mm Hg as being clinically similar enough for meta-analytical pooling. Certain studies used a specific cutoff point to define at-risk vs control instead of comparing 2 ‘double-sided’ ranges such as 140-160 mm Hg vs 120-140 mm Hg. In this case, the comparison was deemed eligible for meta-analysis, as long as the cutoff value was within 10 mm Hg margin of the 100-140 mm Hg baseline range from either side, ie, 90-110 mm Hg for the lower bound (100 ± 10 mm Hg) and 130-150 mm Hg for the upper bound (140 ± 10 mm Hg). Thus, for example, a comparison that used cutoff values of ≤90 mm Hg or ≥130 mm Hg would be included but a study that used cutoff values of ≤80 mm Hg or ≥160 mm Hg^31^ would not be eligible. We excluded data points that compared 2 groups falling within the control range (eg, 110-120 vs 130-140 mm Hg).^27^^,^^30^

**Figure 1:**
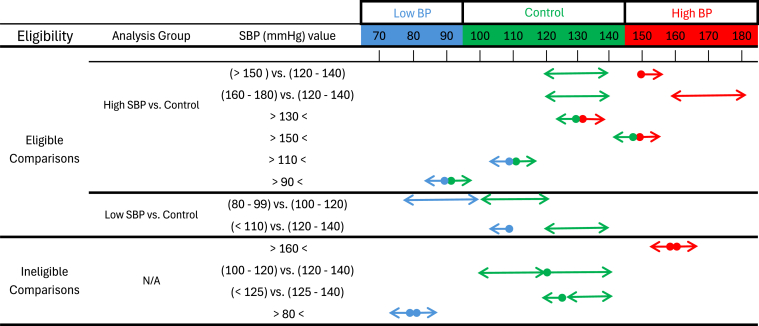
Systolic blood pressure (SBP) meta-analysis eligibility criteria. This figure illustrates the decision-making process for including or excluding studies based on SBP ranges for meta- analysis. The control range was defined as 100-140 mm Hg, and any comparisons involving SBP values outside this range were classified as exposure (low BP and high BP) groups. Studies were eligible for meta-analysis if the SBP comparison involved ≥1 value within 10 mm Hg of the control range (90-110 mm Hg for the lower bound or 130-150 mm Hg for the upper bound). Green arrows represent groups with values that fall within the meta-analysis–defined control range of 100-140 mm Hg. Blue and red arrows indicate groups with values that fall outside the control range by >10 mm Hg, on the lower or higher end, respectively. For a comparison to be eligible, it must include a control group (green) and an exposure group (blue or red). The above comparison groups are examples taken from actual studies included in the meta-analysis, selected here for illustrative purposes. BP, blood pressure; N/A, not applicable.

We illustrated these decision processes in Fig 1. Green arrows represent groups with values that fall within the meta-analysis–defined control range of 100-140 mm Hg. Blue and red arrows indicate groups with values that fall outside the control range by >10 mm Hg, on the lower or higher end, respectively. For a comparison to be eligible, it must include a control group (green) and an exposure group (blue or red). The comparison groups in Fig 1 are examples taken from actual studies included in our meta-analysis, selected for illustrative purposes.

We then examined the risk of all-cause mortality associated with lower SBP levels, also using 100-140 mm Hg as the control condition. We considered combining comparisons for this analysis under 2 scenarios. First, when studies compared two 2-sided ranges, the lower bound of the control condition could not be <90 mm Hg, and the upper bound of the at-risk group could not be >110 mm Hg (using the same ±10 mm Hg margin as mentioned above). Hence, studies comparing 80-90 mm Hg with 91-100 mm Hg or 80-99 mm Hg with 100-120 mm Hg would be eligible, but those comparing 95-115 mm Hg with 115-135 mm Hg would not. (However, we did not identify any studies matching this scenario.) In the second scenario, we combined studies that compared a range within the control condition range of 100-140 mm Hg but used a cutoff point to define the high-risk group, provided that the cutoff value was not ≥110 mm Hg. For example, a study comparing <110 mm Hg with 110-130 mm Hg^37^ would be eligible, but one comparing <120 mm Hg with 120-140 mm Hg^42^ would not.

#### Exposure: DBP

We compared patients with DBP >90 mm Hg and those with DBP ≤90 mm Hg (control condition). We considered any cutoff within a 5 mm Hg margin (ie, 85-95 mm Hg, which is 90 ±5 mm Hg) to be clinically similar enough and eligible for meta-analysis. We excluded comparisons in which the control condition included values outside of this range (eg, 81-88 vs >88 mm Hg)^38^ or the range of values for both the control and risk groups were lower or higher than this range (eg, <67 vs 68-85 mm Hg).^41^

#### Exposure: Presence vs Absence of Hypertension

As shown in Table 1, several studies categorized participants into groups with and without hypertension, typically defined by a combination of elevated BP readings and the use of antihypertensive medications.

#### Statistical Analyses and Data Synthesis for Meta-Analysis

We conducted meta-analyses using Stata version 17.^48^ We used the ‘metan’ command to pool effect sizes across studies and calculate the overall weighted summary estimate with a 95% confidence interval (CI). Heterogeneity was assessed using the *I*^2^ statistic and Cochran’s Q test using a significance α level of 0.05.

To ensure consistency across studies reporting different effect measures, we transformed all odds ratios (ORs) and hazard ratios (HRs) to risk ratios (RRs) for meta-analysis. Only 1 study^38^ reported an unadjusted OR, which we converted to RR using the method proposed by Zhang and Yu.^49^ Most other studies reported HRs, which reflect time-to-event data. However, in the data points we included for the meta-analysis, BP was assessed only at baseline, and the outcome—all-cause mortality—could be considered relatively rare, although follow-up duration varied. In this context, we made a pragmatic decision to harmonize all estimates as RRs, recognizing that HRs and RRs tend to converge numerically under such conditions if the outcome is rare.

We applied a random-effects model, given that in addition to statistical heterogeneity, there were also clinical, methodological, and demographic heterogeneities. For studies that reported multiple but mutually exclusive eligible comparisons (eg, several SBP ranges outside of the control SBP range),^27^^,^^30^^,^^42^ when deemed scientifically appropriate, we first meta-analytically combined outcome measures within each study using a fixed-effect model to generate a single parameter estimate and 95% CI, and then we used that single data point in our model to generate an overall pooled estimate across studies.

When studies reported both adjusted and unadjusted effect sizes, we entered into the meta-analysis RR estimates based on the most complete adjusted multivariable models. The majority of studies applied Cox proportional hazard models. A few studies^11^^,^^22^^,^^41^ also reported Fine and Gray competing risk models, which generated very similar HRs, and, in those instances, we prioritized the Cox model estimates to reduce heterogeneity.

We then performed sensitivity analyses to assess the degree to which model selection could alter the overall pooled estimate.

To assess the robustness of this approach and given that the number of data points for each meta-analysis was small, we conducted sensitivity analyses, including leave-one-out analyses, and reported the range of point estimates under each figure. These analyses allowed evaluation of the influence of individual studies—including the 2 with unadjusted point estimates^32^^,^^38^—on pooled estimates.

We also conducted a sensitivity analysis using a narrower SBP range of 120-140 mm Hg as the control condition due to concerns that the relation between SBP level and mortality is U-shaped and thus some PD patients in the lower SBP range of 100-140 mm Hg are actually at relatively increased risk for all-cause mortality.

## Results

**Figure 2:**
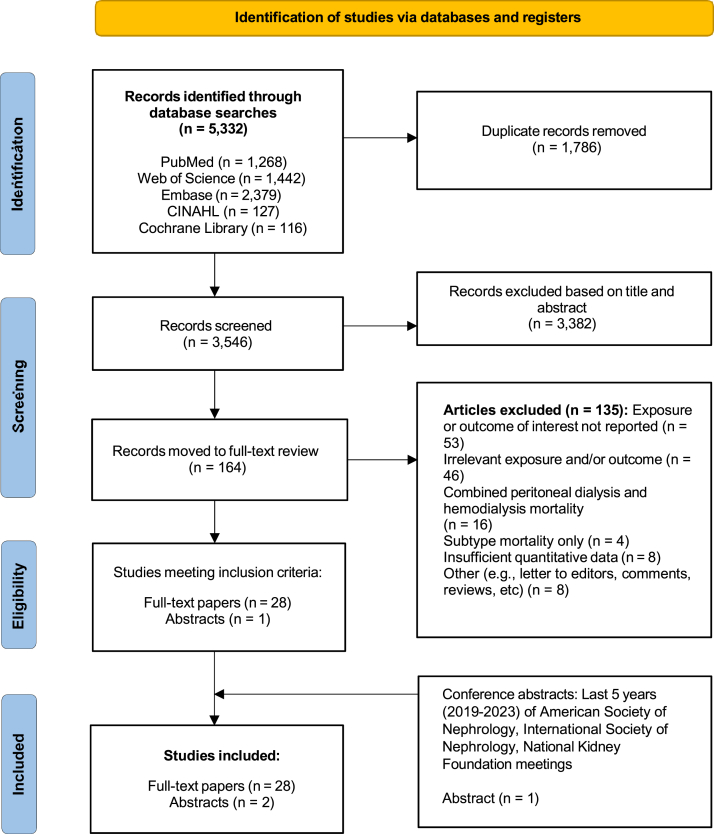
Flowchart of the study selection process for the systematic review and meta-analysis examining the association between blood pressure levels and all-cause mortality in patients with end-stage kidney disease receiving maintenance peritoneal dialysis. The search included 5 databases and conference abstracts from nephrology meetings, covering studies published through April 13, 2023.

Figure 2 shows the results of our search, which yielded 28 full-length articles^11^, ^12^, ^13^^,^^17^, ^18^, ^19^, ^20^, ^21^, ^22^, ^23^, ^24^, ^25^, ^26^, ^27^, ^28^, ^29^, ^30^, ^31^, ^32^, ^33^, ^34^, ^35^, ^36^, ^37^, ^38^, ^39^, ^40^, ^41^ and 1 abstract.^43^ Manual search of major nephrology conference proceedings identified 1 additional abstract.^42^

### Characteristics of Studies Included in the Systematic Review

The characteristics of the thirty included studies are shown in Table 1 and Table S3. The studies span a publication period from 1991 to 2023, with data collection beginning as early as 1979. Twenty-three studies were retrospective,^11^^,^^13^^,^^17^, ^18^, ^19^, ^20^, ^21^, ^22^, ^23^, ^24^^,^^26^^,^^27^^,^^31^^,^^32^^,^^34^^,^^36^, ^37^, ^38^, ^39^, ^40^, ^41^, ^42^, ^43^ whereas 7 were prospective cohorts.^12^^,^^25^^,^^28^, ^29^, ^30^^,^^33^^,^^35^ Sixteen studies were single center,^12^^,^^13^^,^^17^^,^^19^, ^20^, ^21^^,^^24^^,^^25^^,^^28^^,^^32^, ^33^, ^34^, ^35^, ^36^, ^37^^,^^43^ 13 were multicenter,^11^^,^^18^^,^^22^^,^^23^^,^^26^^,^^27^^,^^29^, ^30^, ^31^^,^^38^, ^39^, ^40^, ^41^ and the setting was unclear for 1 study, which was presented only in abstract form.^42^ Twenty-one studies were conducted in Asia: 9 from mainland China,^11^, ^12^, ^13^^,^^21^^,^^23^^,^^25^^,^^34^^,^^37^^,^^41^ 3 from South Korea,^30^^,^^35^^,^^42^ 2 from Taiwan,^32^^,^^33^ and 2 from Thailand^24^^,^^40^ (Table 1). The percentage of female participants ranged from 36% to 68%, and the average age ranged from 42 to 63 years. Although a few studies^11^^,^^24^ included pediatric patients, none focused only on children. Twenty-one studies described their study population as consisting of incident PD patients,^11^, ^12^, ^13^^,^^17^^,^^19^, ^20^, ^21^, ^22^, ^23^, ^24^^,^^26^^,^^27^^,^^29^^,^^31^^,^^34^^,^^36^^,^^37^^,^^39^, ^40^, ^41^, ^42^ 7 with prevalent PD patients,^8^^,^^18^^,^^28^^,^^30^^,^^32^^,^^35^^,^^38^ 1 study included both incident and prevalent PD patients,^25^ and 1 study did not specify.^43^

The risk of bias for the included studies is summarized in Fig S1. Overall, we did not observe any major concerns regarding the selection of exposed and unexposed groups from the same population (D1), the methods used for exposure assessment (D2), or the fact that the outcome of interest was not present at the start of the study (D3). Except for 2 studies,^18^^,^^22^ we also did not observe major concerns with respect to methods used for outcome assessment (D6) and length of follow-up among studies (D7), although our level of confidence for some studies was lower than that for others. However, with the exception of 5 studies,^11^^,^^27^^,^^29^^,^^30^^,^^34^ we observed major limitations concerning the groups’ comparability (D4) and whether prognostic factors were adequately captured (D5). Finally, we were unable to rule out the possibility of existing cointerventions biasing observed associations (D8) in any of the studies.

### Results of the Meta-Analyses

**Figure 3:**
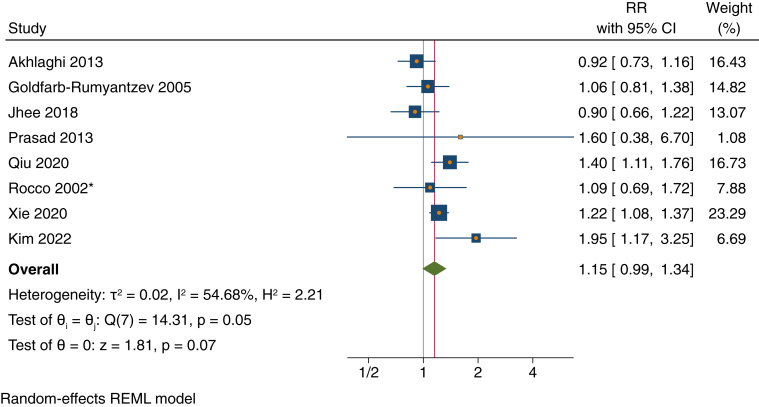
Forest plot of risk ratios (RRs) for high systolic blood pressure (>140 mm Hg) vs control (100-140 mm Hg) for the outcome of all-cause mortality among patients receiving maintenance peritoneal dialysis. Similar results were seen when Xie et al^41^ Fine and Gray model estimate was used. Sensitivity analysis using leave-one-out meta-analysis yielded point estimates (RRs) ranging from 1.11 to 1.20. ∗Rocco et al^38^ estimate was unadjusted. CI, confidence interval; REML, restricted maximum likelihood.

For patients with kidney failure receiving maintenance PD, those with baseline SBP >140 mm Hg had a statistically nonsignificant 15% higher risk of all-cause mortality compared with those with baseline SBP of 100-140 mm Hg (risk ratio [RR], 1.15; 95% CI, 0.99-1.34) (Fig 3).

As reported in Fig 3 legend, results from sensitivity analysis using leave-one-out meta-analysis yielded similar RR point estimates ranging from 1.11 to 1.20. Since the RR estimates from Fine and Gray models were generally not far from the estimates from Cox models (Table 1), results from this and other meta-analyses were similar when we substituted the former for the latter. When we used the narrower SBP control range of 120-140 mm Hg, those with baseline SBP >140 mm Hg had a 19% higher risk of all-cause mortality, but this was also not statistically significant (RR, 1.19; 95% CI, 0.92-1.53) (Fig S2).

Patients with low baseline SBP (<100 mm Hg) had significantly higher mortality rates than those with baseline SBP of 100-140 mm Hg (RR, 1.76; 95% CI, 1.36-2.29) (Fig S3). In the sensitivity analysis using the narrower SBP control range of 120-140 mm Hg, the RR for SBP <120 mm Hg was 1.34 (95% CI, 0.81-2.20) (Fig S4).

**Figure 4:**
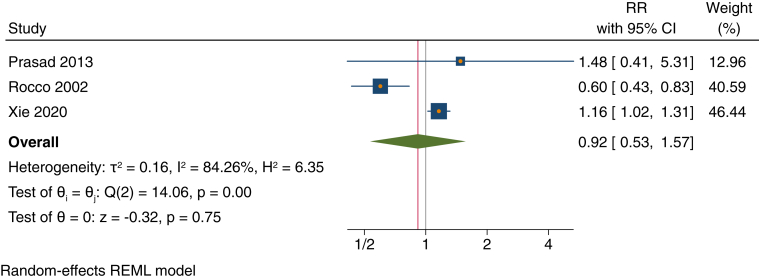
Forest plot of risk ratios (RRs) for high diastolic blood pressure (>90 mm Hg) vs low diastolic blood pressure (≤90 mm Hg) for the outcome of all-cause mortality among patients receiving maintenance peritoneal dialysis. Similar results were seen when Xie et al^41^ Fine and Gray model estimate was used. Sensitivity analysis using leave-one-out meta-analysis yielded point estimates (RRs) ranging from 0.60 to 1.48. CI, confidence interval; REML, restricted maximum likelihood.

For DBP, patients with baseline DBP >90 mm Hg had a slightly lower statistically nonsignificant risk of all-cause mortality than those with baseline DBP ≤90 mm Hg (RR, 0.92; 95% CI, 0.53-1.57) (Fig 4).

**Figure 5:**
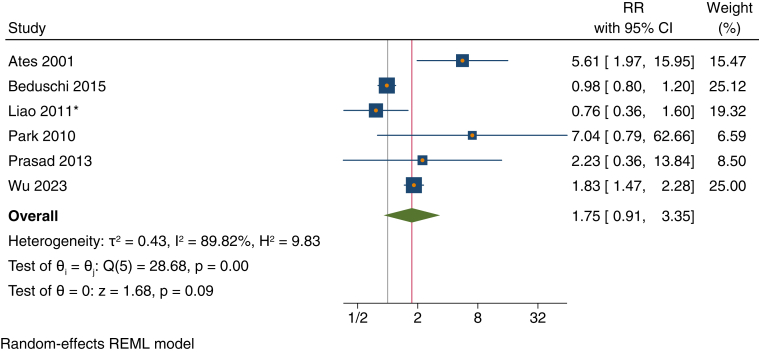
Forest plot of risk ratios (RRs) for presence vs. absence of hypertension for the outcome of all-cause mortality among patients receiving maintenance peritoneal dialysis. Similar results were seen when Beduschi et al^22^ or Wu et al^11^ Fine and Gray model estimates were used. Sensitivity analysis using leave-one-out meta-analysis yielded point estimates (RRs) ranging from 1.32 to 2.15. ∗Liao et al^32^ was unadjusted. CI, confidence interval; REML, restricted maximum likelihood.

Patients with kidney failure with hypertension had a statistically nonsignificant 75% higher risk of all-cause mortality than those without hypertension (RR, 1.75; 95% CI, 0.91-3.35) (Fig 5).

Across all meta-analyses, there was a high degree of heterogeneity (*I*^2^ range, 26%-90%) (Figure 3, Figure 4, Figure 5 and Figs S2-S4).

## Discussion

This systematic review provides a rigorous assessment of the existing evidence base informing clinical practice guidelines for hypertension management in patients with kidney failure receiving maintenance PD. Our analyses suggest that an SBP >140 mm Hg is associated with a statistically nonsignificant 15% higher adjusted risk of all-cause mortality compared with an SBP of 100-140 mm Hg. Additionally, individuals with hypertension had a statistically nonsignificant 75% higher risk of all-cause mortality than those without hypertension. Several factors undermine our level of confidence about the overall quality of the body of evidence.

First, the number of studies devoted to addressing this important clinical question was relatively small. Of the 28 full-length articles, only 9 studies^11^^,^^17^^,^^25^^,^^27^^,^^30^^,^^34^^,^^37^^,^^39^^,^^41^ had BP as the primary exposure, with the rest including BP only as a covariate in multivariable models. Given that we only identified a few data points per each specific outcome for meta-analysis, none exceeding the minimum threshold of 10,^50^ we were unable to statistically assess reporting bias.

Second, there are potential concerns regarding data heterogeneity (consistency) and the relevance of findings (directness) of the evidence. For instance, glomerulonephritis accounted for 50%-60% of the kidney failure cases in several of the more recent, larger studies (which were from Asia).^11^^,^^37^^,^^41^ In contrast, the leading etiology of kidney failure among patients receiving PD in the United States, United Kingdom, and Australia/New Zealand is diabetes.^51^, ^52^, ^53^ Additionally, 100% of patients in some studies^11^^,^^37^ were treated with continuous ambulatory PD, even though automated PD is more commonly used in other parts of the world.^1^^,^^53^ Different clinical practices, drug regimen, prevalence of comorbid conditions, general access to health care, and patient age distribution might also have contributed to the observed high heterogeneity.

Third, studies varied in risk of bias; all were observational, only a few measured exposure and outcome prospectively, and more were single center than multicenter. The large majority of studies were deemed to not have sufficiently captured prognostic factors or cointerventions that may vary by BP categories, thus introducing confounding and other threats to internal validity. Importantly, few studies attempted to address concerns regarding reverse causation. Similar U-shaped associations have been observed in other populations (eg, older individuals), and a key hypothesis emphasized by researchers in other fields is that low SBP serves as a marker of ill health (ie, cause and effect).^54^, ^55^, ^56^ Consistent with this, Afshinnia et al^17^ noted that SBP began to decline around 2 years before death among patients receiving maintenance PD. Analyzing BP as a time-varying exposure exacerbates this problem of reverse causation. In Afshinnia et al,^17^ higher SBP at initiation of PD was associated with a higher crude risk of death, but when treating SBP as a time-varying exposure without adding any other covariates, a U-shaped association emerged. A plausible explanation is that decreasing SBP levels closer to the time of death reflected declining overall health. One approach to address this bias is to exclude deaths that occur relatively soon after ascertainment of initial blood pressure^39^^,^^55^ but this was rarely done. In one of the few examples among PD studies when this was performed, Udayaraj et al^39^ found that higher BP was associated with lower mortality within 6-12 months of starting maintenance PD but with higher mortality after 5 years.

Fourth, with respect to imprecision, the CIs for both key findings (effect of SBP of 100-140 mm Hg and hypertension on all-cause mortality) were relatively wide and included the possibility of a null association, contributing to lowering the level of certainty about the evidence.

We also found no evidence to support targeting DBP <90 mm Hg as DBP above this was not associated with a higher risk of all-cause mortality. Arterial stiffness widens pulse pressure and lowers DBP, which may explain why lower DBP levels may be associated with adverse outcomes.

The strengths of this study include a comprehensive review of an important topic in a population that has not received as much attention as others. For example, a recent Scientific Statement from the American Heart Association on hypertension in patients undergoing maintenance dialysis^57^ focused exclusively on patients receiving hemodialysis and excluded those receiving PD because the authors felt there was insufficient evidence for the latter (personal communication from lead author Dr Nisha Bansal). We followed a rigorous methodology for conducting our systematic review and meta-analysis, including pre-publishing our protocol on PROSPERO. This study identified several key gaps and directions for future research. We believe that future epidemiology studies should include patients from more parts of the world, have larger sample sizes, better control for potential confounding, and better address the issue of reverse causation. Our results also highlighted the great need for randomized clinical trials to inform clinical practice.

Besides the inherent limitations of included studies, several limitations of our review should be acknowledged. Although we thoroughly searched several medical databases and sources of the gray literature, we cannot preclude that publication bias remains. In addition, we did not search for the abstracts from meetings of the International Society for Peritoneal Dialysis or other conferences. We only examined all-cause mortality, not cardiovascular disease events or cardiovascular-specific deaths, which are presumed to be more directly influenced by BP levels. We did not perform stratified analyses for key subgroups, such as incident vs prevalent PD patients or those on continuous ambulatory vs automated PD or adult vs pediatric patients, because either the original studies did not report stratified data or there were insufficient data. The published studies also did not distinguish between patients who were and were not taking BP medications.

In conclusion, the existing body of epidemiologic evidence is insufficient to define optimal BP treatment targets for patients on maintenance PD. Although some findings are not inconsistent with a SBP target of 140 mm Hg, existing studies vary considerably in design, quality, populations, and methodological rigor, limiting their ability to support definitive recommendations. Our findings highlight gaps in the literature and the need for more rigorous observational studies and ideally well-designed randomized trials that explore the impact of targeting different BP levels among patients with kidney failure receiving maintenance PD.^57^, ^58^, ^59^, ^60^
