# Supplementary material for: Blood Pressure Level and Risk of All-Cause Mortality in Patients With Kidney Failure on Maintenance Peritoneal Dialysis: A Systematic Review and Meta-Analysis of Observational Studies
Source: Kidney Med. 2025 Nov 26;8(2):101193. doi: 10.1016/j.xkme.2025.101193 (PMC12864663; doi:10.1016/j.xkme.2025.101193)
Supplement: Supplementary File (PDF) — Figure S1-S3; Table S1-S3. [file mmc1.docx]

**SUPPLEMENTARY MATERIAL**

**Table S1.** Yield of databases

| DATABASE | YIELD |
| --- | --- |
| PubMed | 1,268 |
| Web of Science | 1,442 |
| Embase | 2,379 |
| CINAHL | 127 |
| Cochrane Library | 116 |
| Gross Total | 5,332 |
| Subtract duplicates using EndNote 21 (automated) | 1,289 |
| Subtract duplicates using Rayyan (manual) | 497 |
| Net Total | 3,546 |

**Table S2.** Search strategy by database

| DATABASE | SEARCH STRATEGY |
| --- | --- |
| PubMed | (“blood pressure” OR “Blood Pressure”[Mesh] OR hypertension OR "Hypertension"[Mesh]) AND (“peritoneal dialysis” OR "Peritoneal Dialysis"[Mesh]) AND (mortality OR “Mortality”[Mesh] OR death OR "Death"[Mesh] OR “adverse event*” OR “adverse occurrence*” OR  surviv* OR “Survival”[Mesh]) |
| Web of Science | (“blood pressure” OR hypertension) AND “peritoneal dialysis” AND (mortality OR death OR “adverse event*” OR “adverse occurrence*” OR  surviv*) |
| Embase | ('blood pressure'/exp OR 'blood pressure' OR 'hypertension'/exp OR hypertension) AND ('peritoneal dialysis'/exp OR 'peritoneal dialysis') AND ('mortality'/exp OR 'mortality' OR 'death'/exp OR 'death' OR 'adverse event'/exp OR 'adverse event' OR 'adverse occurrence*' OR 'survival'/exp  OR surviv*) |
| CINAHL | (“blood pressure” OR hypertension) AND “peritoneal dialysis” AND (mortality OR death OR “adverse event*” OR “adverse occurrence*” OR  surviv*) |
| Cochrane Library | (“blood pressure” OR hypertension) AND “peritoneal dialysis” AND (mortality OR death OR “adverse event*” OR “adverse occurrence*” OR  surviv*) |

**Table S3.** Inclusion and exclusion criteria as reported by study authors

| **Author & Year** | **Inclusion Criteria** | **Exclusion Criteria** |
| --- | --- | --- |
| **Afshinnia et al. 2016** | 18 years of age or older, initiation of renal replacement therapy with PD as the modality in patients with ESKD from any etiology, at least 3 months of follow-up available after the PD start, and having routinely performed serial echocardiography  examinations during follow-up | Exclusion criteria were recovery from kidney failure leading to termination of PD, transitioning to HD or kidney transplantation, as well as transferring of care to another outpatient unit within 3 months after dialysis initiation. |
| **Akhlaghi et al. 2013** | All patients with renal failure who had been treated by CAPD and followed for 3 months; The data were obtained, retrospectively, from a total of 20 medical centers all over Iran who registered in  CAPD Database | Not reported |
| **Ates et al. 2001** | Patients who survived more than six months on PD and had started their PD treatment between 1992  and 1996 | Not reported |
| **Ates et al. 2005** | Patients who survived more than 3 months on PD and who were monitored for 3 years from the time of starting of PD. | Patients with chronic infection, amyloidosis, vasculitis, malignancy, or chronic liver disease were excluded from the study. Patients with fewer than four serum CRP  measurements were also excluded. |
| **Bao 2019** | Incident PD patients who initiated PD from 20-Sep-2005 to 05-Feb- 2008. | (1) persistent hypotension  blood pressure (SBP) < 90 mmHg,  (2) paralytic or cognitive impairment following cerebrovascular event, (3) absence of femoral or radial pulse,  (4) an unwillingness to participate,  (5) clinical unstable like peritonitis, acute heart failure, (6) had malignant disease |
| **Beduschi et al. 2015** | Incident adult patients who remained at least 90 days in PD | Those who switched modality at any time during the follow-up period.  Patients on PD for less than 90 days. |
| **Cao et al. 2015** | ≥18 year old or older, either gender, CAPD ≥ 3 months, explicit time of OD catheter implantation stated, baseline lab tests completed within the 3 months before PD  placement, clear outcome time and circumstances, and followed for | Non-CAPD patients, cancer, severe complications in the heart, brain or other organs, missing basic information, and incomplete baseline data |

|  | one year or had end events within  one year |  |
| --- | --- | --- |
| **Chaichaya et al. 2020** | All patients at least 15 years old  with stage-5 CKD who started PD | Patients with incomplete data  required for analysis were excluded |
| **Chen et al. 2022** | Adult incident PD patients from Huashan Hospital, Fudan University, China | Patients were excluded if they were transferred from hemodialysis or failed renal transplantation. Patients exhibited an ongoing infection, neoplasia, or unstable cardiovascular disease with a life expectancy of less  than 6 months were also excluded. |
| **Dai et al. 2020** | Prevalent adult PD patients from Huasan Hospital, Fudan University, enrolled from March 2001 to July  2018 | Excluded if they had an ongoing infection or were clinically unstable, with a life expectancy of fewer than  6 months |
| **Fang et al. 2009** | All patients aged 18 years or older that commenced PD between 1 January 2000 and 31 December  2005 at the University Health Network, Toronto | Not reported |
| **Goldfarb- Rumyantzev et al. 2005** | Patients with ESRD at the time of initiation of the DMMS Wave 2 study, who survived >90 days since the onset of ESRD, were included in this analysis (time at risk for survival analysis therefore begins  90 days after starting dialysis). | The following exclusion criteria were applied: (i) patients<18 years of age at the study start [2]; (ii) those who recovered renal function and no longer required dialysis; and (iii) patients for whom BP measurements  were not available. |
| **Iliescu et al. 2002** | Prevalent patients on PD at Kingston General Hospital, Kingston, Ontario, Canada | Patients were excluded if they chose not to participate, involved in another study, and the sample not  suitable for Lp(a) assay |
| **Jager et al. 1999** | End-stage renal disease patients who were older than 18 years when starting chronic PD, who had never received renal replacement therapy in the past, and who had survived the first three months on dialysis were eligible for the study.  Included consecutive patients who started PD between October 1, 1993, and April 1, 1995. | Not reported |
| **Jhee et al. 2018** | Data were retrieved from the Clinical Research Center for End- Stage Renal Disease to which patients undergoing prevalent  dialysis (both HD and PD) were | Excluded patients who met the following criteria: 1) age <18 years,  2) SBP <70 or >210 mmHg, 3)  scheduled to undergo kidney |

|  | prospectively enrolled from 2009 to  2014 in South Korea | transplantation, and 4) with missing  data during follow-up |
| --- | --- | --- |
| **Kemperman et al. 1991** | Patients with diabetes mellitus and end stage renal disease due to diabetic nephropathy started on CAPD between July 1979 and July 1989 at three hospitals in the  Netherlands | Excluded patients who were treated for less than 3 months on PD |
| **Liao et al. 2011** | Patients from a medical center in North Taiwan who began PD between January 1999 and December 2005 | 64 patients were excluded by the following criteria: <18 years of age (n = 7), lack of initial biochemical data (n = 9), failed renal transplant (n  = 4) and discontinuation of PD treatment within 3 months (n = 44) |
| **Liu et al. 2008** | The inclusion criteria were regular PD for at least 3 months before enrollment and clinical stability for at least 3 months before entry without infectious or other active  diseases. | The only exclusion criterion was a history of renal transplantation or maintenance of HD >3 months prior to the study. |
| **Lyu et al. 2019** | 1) Aged 18 years and above; 2) No history of hemodialysis or transplantation; 3) Received peritoneal dialysis for 3 months or more; 4) At least 2 outpatient blood pressure monitoring records within  3 months after the start of peritoneal dialysis. | Not reported |
| **Park et al. 2010** | Prevalent CAPD patients, who had maintained PD for >3 months from a single Korean dialysis center and followed at Yonsei University Health System in Seoul, Korea | Excluded patients who were younger than 18 years of age, had overt infections during the last 3 months prior to study enrollment or had a history of malignancy or other chronic inflammatory disease (e.g. rheumatoid arthritis or systemic lupus erythematosus). To reduce confounding effects from glucose and lipid metabolism, we also  excluded diabetic patients. |
| **Prasad et al. 2013** | End stage renal disease patients who have been initiated on PD from November 2004 to December 2011 at the tertiary care institute  and non-diabetic PD patients who were on PD for at least 3 months | Diabetic patients, patients of less than 12 years of age, patients using icodextrin fluid, and those who could not continue PD for at least 3 months |

| **Qiu et al. 2020** | Eligible participants were over 18 years of age and undergoing continuous ambulatory peritoneal dialysis (CAPD) treatment for >3 months | Excluded those with a history of kidney transplantation, HD longer than 3 months, malignant disease, or missing serum sodium or BP data at  baseline |
| --- | --- | --- |
| **Rocco et al. 2002** | Adult PD patients alive on December 31, 1996, identified by CMS for inclusion in the 1997 PD CIP cohort; Analysis was restricted to patients who had not received hemodialysis at any  time during the study period, but  who had been on PD for part or all of the 6-month reporting interval. | Not reported |
| **Udayaraj et al. 2009** | Incident adult (18 years) patients starting RRT between January 1, 1997, and December 31, 2004, and on PD therapy at day 180 from the start of RRT in renal units in England and Wales reporting to the  UKRR | Missing ethnicity, missing cause of end-stage renal, missing BP or other baseline laboratory data in the first 2 quarters of RRT |
| **Vejakama et al. 2013** | Patients at 82 general hospitals aged 15 years or older were eligible if they met the following criteria: Firstly initiated CAPD and participated in the CAPD first- policy from January 2008 to April2011, survived more than 1 month after initiating CAPD, and had at least 1tKt/V during the studied period. | Patients were ineligible if they had the following criteria: on CAPD due to acute renal failure, aged > 100 years, tKt/V < 0.5 or > 5, tCrcl < 10 or > 400 L/week/1.73 m2,serum albumin level < 0.3 or > 6 g/dl, hemoglobin level< 3 or > 20 g/dl, urine volume < 0 or > 4,000 ml, ultra-filtration (UF) volume <  −2,000 or > 4,000 ml, systolic blood pressure (SBP) < 40 or > 300 mmHg, or diastolic blood pressure (DBP) < 10 or > 200 mmHg. |
| **Wu et al. 2023** | Incident CAPD patients from five PD centers in three provinces in  China | No patient was excluded from this study. |
| **Xie et al. 2020** | Incident cohort of ESRD patients (>18 years) solely on peritoneal dialysis therapy, who had a follow- up of more than 3 months and had at least two or more outpatient blood pressure measurements  within 3 months after the initiation of peritoneal dialysis therapy, from | Patients with missing baseline data and records |

|  | 1 January 2008 to 31 December  2016 |  |
| --- | --- | --- |
| **Xu et al. 2021** | The inclusion criteria were: age ≥ 18 years; PD treatment ≥ 3 months. | The exclusion criteria were: out-of- hospital catheterization, previous history of kidney transplantation; previous dialysis ≥3 months; CVD occurrence within 3 months; patients with rheumatic or organic heart disease; patients with chronic liver disease and severe liver dysfunction; those who have had blood transfusion within 3 months before the study; those with incomplete clinical data; those who have had a history of thyroid disease or hypothalamo-pituitary disorders now or in the past; determined thyroid hormone levels (within 1 month), and took medicines that may affect the secretion and metabolism of  thyroid hormone. |
| **Kim et al. 2022 (abstract)** | Incident PD patients who had more  than 6 months’ follow-up between 2000 to 2019 | Not reported |
| **Kurahashi et al. 2020 (abstract)** | Patients undergoing PD managed in the hospital from April 2010 to  December 2017 were participated in this study | Not reported |

**
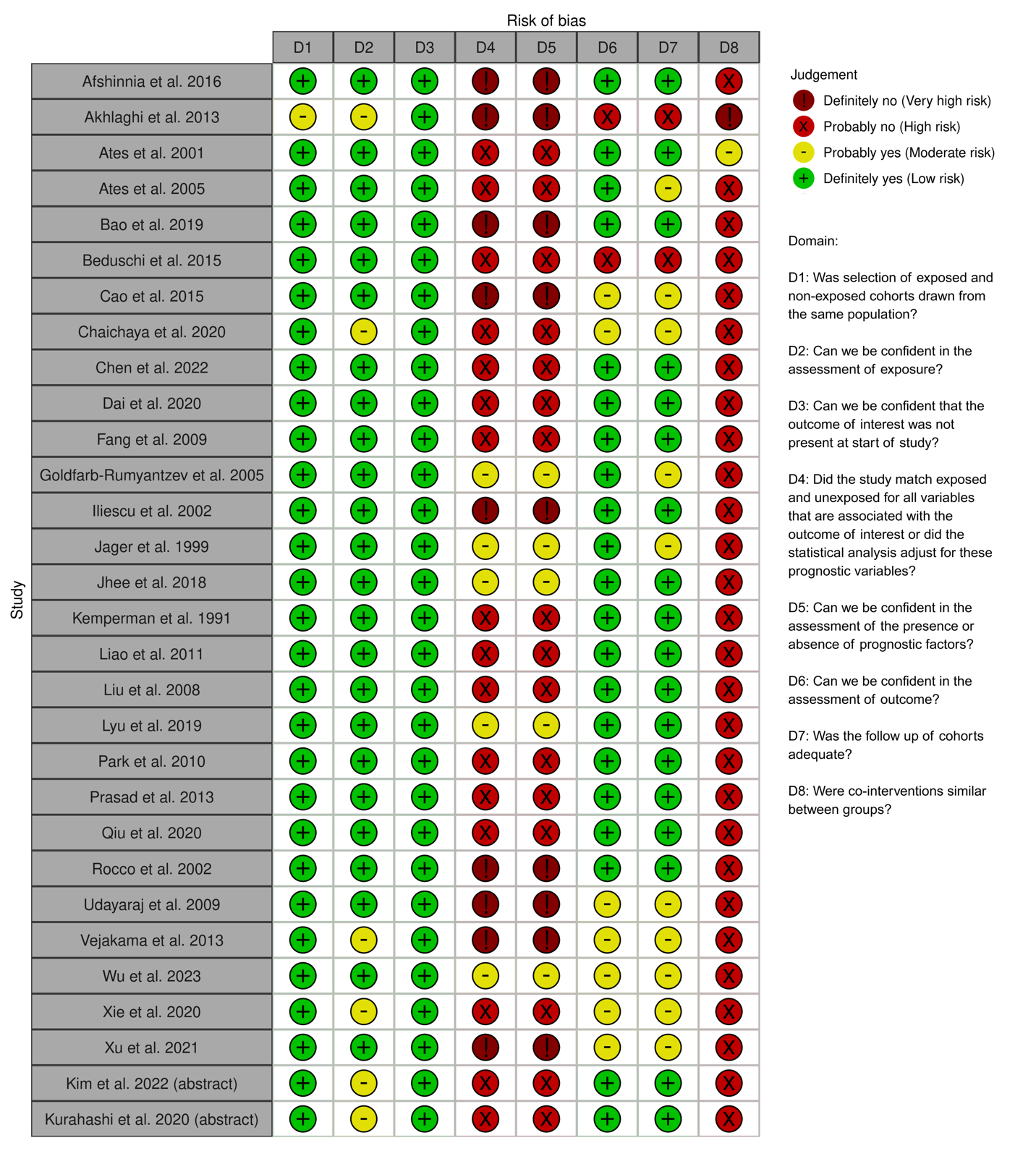
**

**Figure S1.** Risk of bias assessment across eight domains using the risk of bias tool for cohort studies developed by the Clinical Advances Through Research and Information Translation (CLARITY) group at McMaster University (University CGaM. Tool to Assess Risk of Bias in Cohort Studies. <https://www.distillersr.com/info/tool-to-assess-risk-of-bias-in-cohort-studies-ty>). The domains assess selection, exposure, outcome presence at study entry, variable matching/adjustment, prognostic factors, outcome assessment, adequacy of follow-up, and co-interventions. Each study is rated on a color scale ranging from low risk (green) to very high risk (red). Visualization generated using the robvis tool.

**
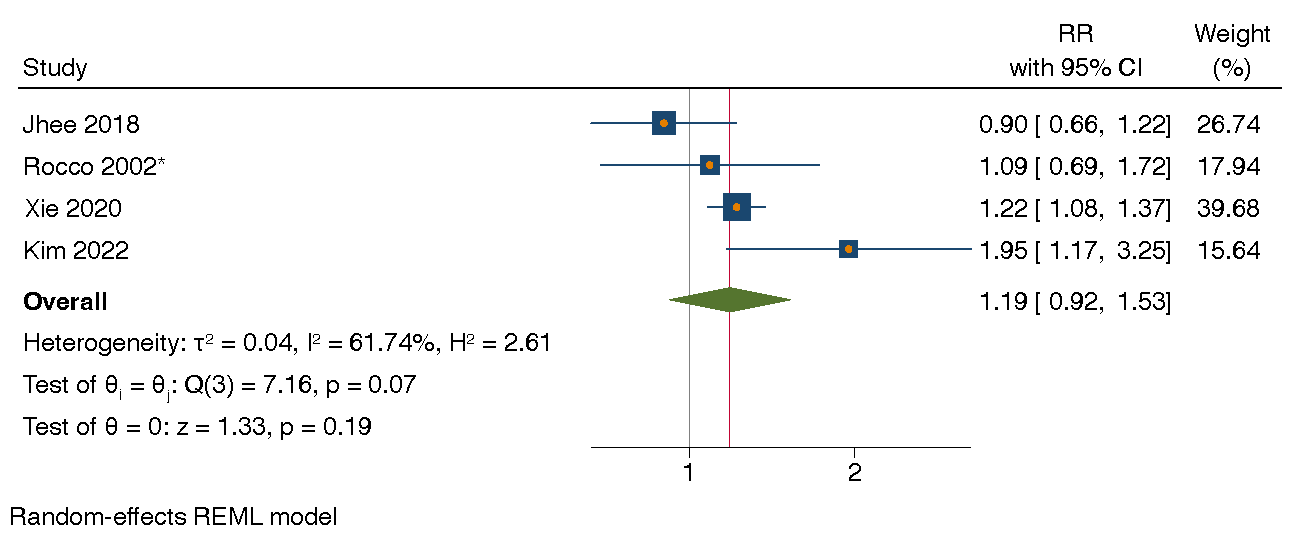
**

**Figure S2:** Forest plot of risk ratios for high systolic blood pressure (>140 mmHg) vs. narrowed control range (120–140 mmHg) for outcome of all-cause mortality among patients on maintenance peritoneal dialysis. Similar results were seen when Xie 2020 Fine & Gray model estimate was used. Sensitivity analysis using leave-one-out meta-analysis yielded point estimates (RR) ranging from 1.10–1.28. *Rocco 2002 estimate was unadjusted.

**
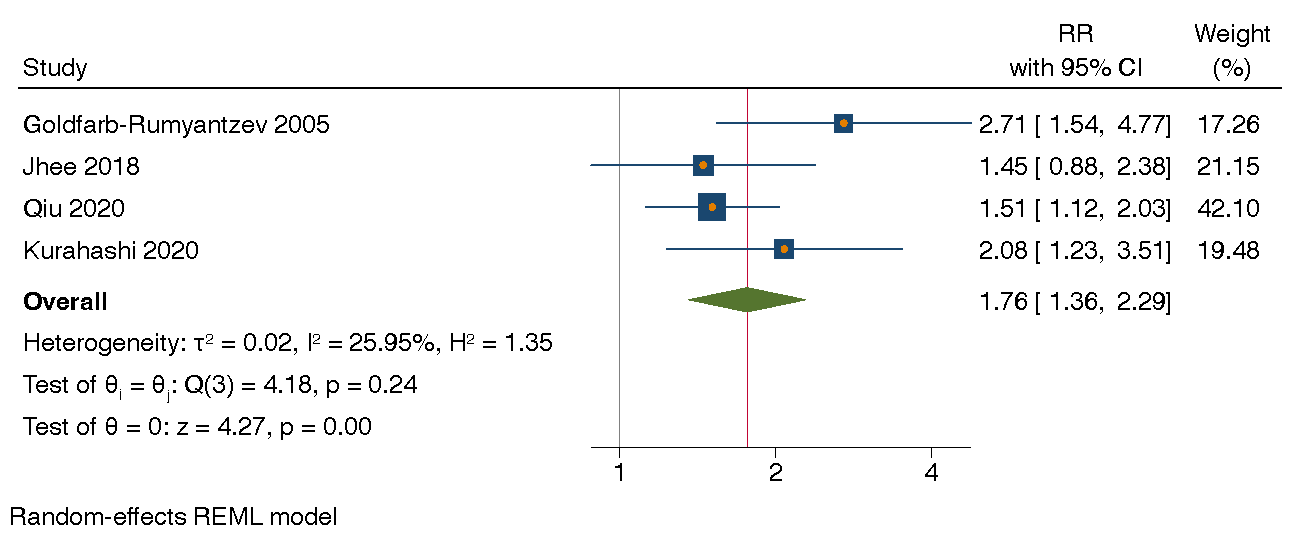
**

**Figure S3**. Forest plot of risk ratios for low systolic blood pressure (<100 mmHg) vs. control range (100–140 mmHg) for outcome of all-cause mortality among patients on maintenance peritoneal dialysis. Sensitivity analysis using leave-one-out meta-analysis yielded point estimates (RR) ranging from 1.59–1.98.

**
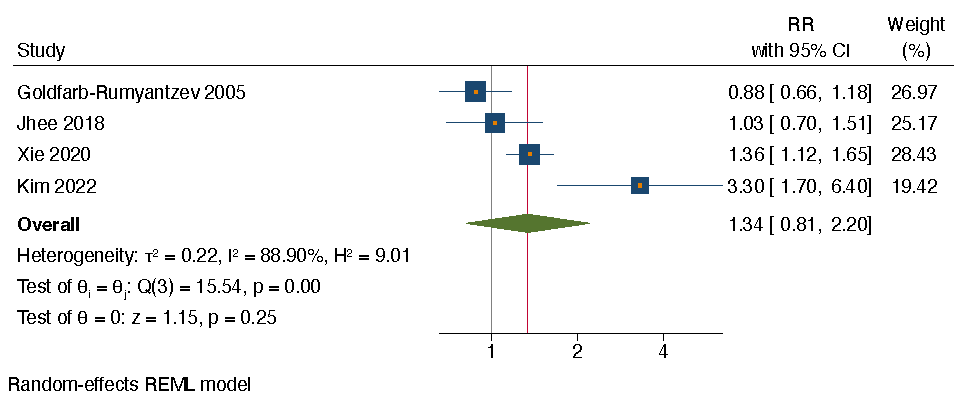
**

**Figure S4:** Forest plot of risk ratios for low systolic blood pressure (<140 mmHg) vs. narrowed control range (120–140 mmHg) for outcome of all-cause mortality among patients on maintenance peritoneal dialysis. Similar results were seen when Xie 2020 Fine & Gray model estimate was used. Sensitivity analysis using leave-one-out meta-analysis yielded point estimates (RR) ranging from 1.10–1.57.

**SUPPLEMENTARY REFERENCES***

**Articles screened at the full text level categorized by their reasons for inclusion or exclusion*

# Included: Association of blood pressure and risk of all-cause mortality in patients with kidney failure on peritoneal dialysis (n = 30)

1. Afshinnia F, Zaky ZS, Metireddy M, Segal JH. Reverse Epidemiology of Blood Pressure in Peritoneal Dialysis Associated with Dynamic Deterioration of Left Ventricular Function. Perit Dial Int. 2016-3 2016;36(2):154-62. doi:doi:10.3747/pdi.2014.00264
2. Akhlaghi AA, Najafi I, Mahmoodi M, Shojaee A, Yousefifard M, Hosseini M. Survival analysis of Iranian patients undergoing continuous ambulatory peritoneal dialysis using cure model. J Res Health Sci. 2013;13(1):32-6. doi:doi:
3. Ateş K, Ateş A, Ekmekçi Y, Nergizoglu G. The time course of serum C-reactive protein is more predictive of mortality than its baseline level in peritoneal dialysis patients. Perit Dial Int. 2005;25(3):256-68. doi:doi:
4. Ateş K, Nergizoğlu G, Keven K, et al.. Effect of fluid and sodium removal on mortality in peritoneal dialysis patients. Kidney Int. 2001;60(2):767-76. doi:doi:10.1046/j.1523- 1755.2001.060002767.x
5. Bao W, Wang F, Tang W. Aortic-brachial stiffness mismatch and mortality in peritoneal dialysis patients. Kidney and Blood Pressure Research. 2019;44(1):123-132. doi:doi:10.1159/000498876
6. Beduschi GD, Figueiredo AE, Olandoski M, et al.. Automated Peritoneal Dialysis Is Associated with Better Survival Rates Compared to Continuous Ambulatory Peritoneal Dialysis: A Propensity Score Matching Analysis. Plos One. 2015;10(7)doi:doi:10.1371/journal.pone.0134047
7. Cao XY, Zhou JH, Cai GY, et al.. Predicting one-year mortality in peritoneal dialysis patients: An analysis of the china peritoneal dialysis registry. International Journal of Medical Sciences. 2015;12(4):354-361. doi:doi:10.7150/ijms.11694
8. Chaichaya N, Thinkhamrop B, Tatiyanupanwong S, et al.. Comparison of all-cause mortality and technique failure between early-late and very late start peritoneal dialysis: A retrospective cohort study. Medico-Legal Update. 2020;20(4):1771-1778. doi:doi:10.37506/mlu.v20i4.2097
9. Chen Y, Dai S, Ge X, et al.. Prognostic values of left ventricular mass index progression in incident peritoneal dialysis patients : a prospective cohort study. BMC Nephrol. 2022;23(1):200. doi:doi:10.1186/s12882-022-02831-6
10. Dai S, Chen Y, Shang D, et al.. Association of Ambulatory Blood Pressure with All-Cause Mortality and Cardiovascular Outcomes in Peritoneal Dialysis Patients. Kidney Blood Press Res. 2020;45(6):890-899. doi:doi:10.1159/000510298
11. Fang W, Yang X, Bargman JM, Oreopoulos DG. Association between pulse pressure and mortality in patients undergoing peritoneal dialysis. Perit Dial Int. 2009;29(2):163-70.
12. Goldfarb-Rumyantzev AS, Baird BC, Leypoldt JK, Cheung AK. The association between BP and mortality in patients on chronic peritoneal dialysis. Nephrol Dial Transplant. 2005;20(8):1693-701. doi:doi:10.1093/ndt/gfh856
13. Iliescu EA, Marcovina SM, Morton AR, Lam M, Koschinsky ML. Apolipoprotein(a) phenotype and lipoprotein(a) level predict peritoneal dialysis patient mortality. Perit Dial Int. 2002;22(4):492-9. doi:doi:
14. Jager KJ, Merkus MP, Dekker FW, et al.. Mortality and technique failure in patients starting chronic peritoneal dialysis: results of The Netherlands Cooperative Study on the Adequacy of Dialysis. NECOSAD Study Group. Kidney Int. 1999;55(4):1476-85. doi:doi:10.1046/j.1523- 1755.1999.00353.x
15. Jhee JH, Park J, Kim H, et al.. The Optimal Blood Pressure Target in Different Dialysis Populations. Sci Rep. 2018;8(1):14123. doi:doi:10.1038/s41598-018-32281-w
16. Kemperman FA, van Leusen R, van Liebergen FJ, et al.. Continuous ambulatory peritoneal dialysis (CAPD) in patients with diabetic nephropathy. Neth J Med. 1991;38(5):236-45. doi:doi:
17. Kim J-k, Lee DH, Kim S, Kim SG. Impact of Blood Pressure on Mortality in Patients Undergoing Peritoneal Dialysis [abstract]. presented at: American Society of Nephrology Kidney Week 2022; November 3-6, 2022; Orland, FL.
18. Kurahashi M, Harada K, Kanai H. THE ASSOCIATION WITH LOWER SYSTOLIC BLOOD PRESSURE AND ALL-CAUSE MORTALITY IN PATIENTS UNDERGOING PERITONEAL

DIALYSIS. Nephrology Dialysis Transplantation. 2020;35:1472-1472. doi:doi:

1. Liao CT, Kao TW, Chou YH, et al.. Associations of metabolic syndrome and its components with cardiovascular outcomes among non-diabetic patients undergoing maintenance peritoneal dialysis. Nephrology Dialysis Transplantation. 2011;26(12):4047-4054. doi:doi:10.1093/ndt/gfr175
2. Liu JH, Chen CC, Wang SM, et al.. Association between pulse pressure and 30-month all-cause mortality in peritoneal dialysis patients. Am J Hypertens. 2008;21(12):1318-23. doi:doi:10.1038/ajh.2008.286
3. Lyu D, Xie X, Zhang X, Chen J. [Associations of mean arterial pressure levels with mortality in patients with peritoneal dialysis]. Zhejiang Da Xue Xue Bao Yi Xue Ban. 2019;48(2):180-185. doi:doi:10.3785/j.issn.1008-9292.2019.04.09
4. Park JT, Chang TI, Kim DK, et al.. Metabolic syndrome predicts mortality in non-diabetic patients on continuous ambulatory peritoneal dialysis. Nephrol Dial Transplant. 2010;25(2):599- 604. doi:doi:10.1093/ndt/gfp498
5. Prasad N, Sinha A, Gupta A, et al.. Effect of metabolic syndrome on clinical outcomes of non- diabetic peritoneal dialysis patients in India. Nephrology (Carlton). 2013;18(10):657-64. doi:doi:10.1111/nep.12124
6. Qiu Y, Ye H, Fan L, et al.. Serum Sodium Modifies the Association of Systolic Blood Pressure with Mortality in Peritoneal Dialysis Patients. Kidney Blood Press Res. 2020;45(6):916-925. doi:doi:10.1159/000510478
7. Rocco MV, Frankenfield DL, Prowant B, Frederick P, Flanigan MJ. Risk factors for early mortality in U.S. peritoneal dialysis patients: impact of residual renal function. Perit Dial Int. 2002;22(3):371-9. doi:doi:
8. Udayaraj UP, Steenkamp R, Caskey FJ, et al.. Blood pressure and mortality risk on peritoneal dialysis. Am J Kidney Dis. 2009;53(1):70-8. doi:doi:10.1053/j.ajkd.2008.08.030
9. Vejakama P, Thakkinstian A, Ingsathit A, Dhanakijcharoen P, Attia J. Prognostic factors of all- cause mortalities in continuous ambulatory peritoneal dialysis: a cohort study. BMC Nephrol. 2013;14:28. doi:doi:10.1186/1471-2369-14-28
10. Wu J, Zhan X, Wen Y, et al.. Preexisting Cardiovascular Disease, Hypertension, and Mortality in Peritoneal Dialysis. Reviews in Cardiovascular Medicine. 2023;24(1)doi:doi:10.31083/j.rcm2401030
11. Xie X, Lv D, Zheng H, Zhang X, Han F, Chen J. The associations of blood pressure parameters with all-cause and cardiovascular mortality in peritoneal dialysis patients: a cohort study in China. J Hypertens. 2020;38(11):2252-2260. doi:doi:10.1097/hjh.0000000000002526
12. Xu L-C, Zhou F-F, Li M, et al.. The Correlation Between Low Serum T3 Levels and All-Cause and Cardiovascular Mortality in Peritoneal Dialysis Patients. Therapeutics & Clinical Risk Management. 2021;17:851-861. doi:doi:10.2147/TCRM.S324672

# Excluded: Exposure or outcome of interest not reported (n = 53)

1. Abe M, Hamano T, Hoshino J, et al. Predictors of outcomes in patients on peritoneal dialysis: A 2-year nationwide cohort study. Sci Rep. 2019;9(1):3967. doi:doi:10.1038/s41598-019-40692-6
2. Bäck C, Hornum M, Møller CJH, Olsen PS. Cardiac surgery in patients with end-stage renal disease on dialysis. Scandinavian Cardiovascular Journal. 2017;51(6):334-338. doi:doi:10.1080/14017431.2017.1384565
3. Balaskas EV, Yuan ZY, Gupta A, et al. Long-term continuous ambulatory peritoneal dialysis in diabetics. Clin Nephrol. 1994;42(1):54-62. doi:doi:
4. Bammatter F, Keusch G, Schiffl H. Continuous ambulatory peritoneal dialysis. Schweizerische Medizinische Wochenschrift. 1980;110(18):689-697. doi:doi:
5. Bostom AG, Shemin D, Verhoef P, et al. Elevated fasting total plasma homocysteine levels and cardiovascular disease outcomes in maintenance dialysis patients - A prospective study. Arteriosclerosis Thrombosis and Vascular Biology. 1997;17(11):2554-2558. doi:doi:10.1161/01.Atv.17.11.2554
6. Brogan D, Kutner NG, Flagg E. Survival differences among older dialysis patients in the southeast. American Journal of Kidney Diseases. 1992;20(4):376-386. doi:doi:10.1016/S0272- 6386(12)70302-5
7. Cader RA, Gafor HA, Mohd R, et al. Blood Pressure Profile In Continuous Ambulatory Peritoneal Dialysis Patients. Excli Journal. 2012;11:116-124.
8. Chang JH, Sung JY, Ahn SY, et al. Hemodialysis leads to better survival in patients with diabetes or high comorbidity, compared to peritoneal dialysis. Tohoku J Exp Med. 2013;229(4):271-7. doi:doi:10.1620/tjem.229.271
9. Chien C-C, Wang J-J, Sun Y-M, et al.. Long-term survival and predictors for mortality among dialysis patients in an endemic area for chronic liver disease: a national cohort study in Taiwan. BMC Nephrology. 2012;13(1):43-43. doi:doi:10.1186/1471-2369-13-43
10. Choi SJ, Obi Y, Ko GJ, et al.. Comparing Patient Survival of Home Hemodialysis and Peritoneal Dialysis Patients. Am J Nephrol. 2020;51(3):192-200. doi:doi:10.1159/000504691
11. Ferńandez MA, Ortiz AM, Valenzuela M, Morales RA. Peritoneal dialysis in chronic renal failure patients over 65 years of age. Adv Perit Dial. 2004;20:128-31. doi:doi:
12. Foley RN, Parfrey PS, Harnett JD, Kent GM, Murray DC, Barre PE. Impact of hypertension on cardiomyopathy, morbidity and mortality in end-stage renal disease. Kidney Int. 1996;49(5):1379-85. doi:doi:10.1038/ki.1996.194
13. Ge LN, Fang W, Zhang L, et al.. Risk factors of pulse pressure and association of pulse pressure with mortality in patients undergoing peritoneal dialysis. Journal of Shanghai Jiaotong University (Medical Science). 2012;32(3):307-311. doi:doi:10.3969/j.issn.1674- 8115.2012.03.015
14. Georgianos PI, Vaios V, Zebekakis PE, Liakopoulos V. The Relation of Clinic and Ambulatory BP with the Risk of Cardiovascular Events and All-Cause Mortality among Patients on Peritoneal Dialysis. J Clin Med. 2021;10(11)doi:doi:10.3390/jcm10112232
15. Ghonimi TA, Hamad A, Iqbal Z, et al.. Mortality of dialysis patients in Qatar: A retrospective epidemiologic study. Qatar Medical Journal. 2021;2021(1)doi:doi:10.5339/QMJ.2021.2
16. Goldfarb-Rumyantzev AS, Baird BC, Leypoldt JK, Habib AN, Cheung AK. Association between blood pressure and mortality in patients on chronic peritoneal dialysis. American Journal of Kidney Diseases. 2005;45(4):A27-A27. doi:doi:
17. Goldfarb-Rumyantzev AS, Pappas L, Stone MB, White G, Leypoldt JK, Cheung AK. Blood pressure (BP) control and mortality risk in patients with ESRD on peritoneal dialysis (PD). Journal of the American Society of Nephrology. 2002;13:614A-615A. doi:doi:
18. Gomez AT, Kiberd BA, Royston JP, et al.. Comorbidity burden at dialysis initiation and mortality: A cohort study. Canadian Journal of Kidney Health and Disease. 2015;2(1)doi:doi:10.1186/s40697-015-0068-3
19. Gu WL, Yang X, Yi CY, Yu XQ. Prevalence of metabolic syndrome and its risk factors in patients with continuous ambulatory peritoneal dialysis in South China. Clinical Nephrology. 2013;80(2):114-120. doi:doi:10.5414/cn107849
20. Gunawan A, Sakti PT. Five-Year Survival Rate of Patients with End-Stage Renal Disease on Continuous Ambulatory Peritoneal Dialysis (CAPD) at Malang CAPD Center, Indonesia. Acta Med Indones. 2023;55(1):4-9. doi:doi:
21. Habib A, Durand AC, Brunet P, et al.. [Comparison of peritoneal dialysis and hemodialysis survival in Provence-Alpes-Côte d'Azur]. Nephrol Ther. 2016;12(4):221-8. doi:doi:10.1016/j.nephro.2016.01.015
22. Hüting J, Schütterle G. Cardiovascular factors influencing survival in end-stage renal disease treated by continuous ambulatory peritoneal dialysis. Am J Cardiol. 1992;69(1):123-7. doi:doi:10.1016/0002-9149(92)90687-t
23. Io H, Suzuki Y. Strategy for Prevention of Left Ventricular Remodeling in Predialysis and Dialysis Patients. Contrib Nephrol. 2018;196:13-21. doi:doi:10.1159/000485691
24. Kobus G, Malyszko J, Mysliwiec M, Bachorzewska-Gajewska H. Hypertension in patients treated with renal replacement therapy. Arterial Hypertension. 2009;13(2):114-119. doi:doi:
25. Koc Y, Unsal A, Ahbap E, Sakaci T, Yilmaz M. Clinical Outcome Of Diabetic Peritoneal Dialysis Patients And Evaluation Of Factors Affecting Mortality: A Single Centre's Experience From Turkey. Journal of Renal Care. 2011;37(2):94-100. doi:doi:10.1111/j.1755-6686.2011.00218.x
26. Kuroki Y, Hori K, Tsuruya K, et al.. Association of blood pressure after peritoneal dialysis initiation with the decline rate of residual kidney function in newly-initiated peritoneal dialysis patients. Plos One. 2021;16(7)doi:doi:10.1371/journal.pone.0254169
27. Lawal CO, Soyibo AK, Frankson A, Barton EN. Characteristics, complications and outcome of patients treated with automated peritoneal dialysis at the Peritoneal Dialysis Unit, University Hospital of the West Indies. West Indian Med J. 2010;59(3):312-8. doi:doi:
28. Lei G, Feng X, Wang X, et al.. Coexistence of diabetes mellitus and pre-existing cardiovascular disease and mortality in Chinese patients on peritoneal dialysis. BMC Nephrol. 2022;23(1):68. doi:doi:10.1186/s12882-022-02702-0
29. Lu R, Estremadoyro C, Chen X, et al.. Hemodialysis versus peritoneal dialysis: an observational study in two international centers. Int J Artif Organs. 2017:0. doi:doi:10.5301/ijao.5000656
30. Maitra S, Burkart J, Fine A, et al.. Patients on chronic peritoneal dialysis for ten years or more in North America. Perit Dial Int. 2000;20:S127-33. doi:doi:
31. McGregor D, Buttimore A, Robson R, Little P, Morton J, Lynn K. Thirty years of universal home dialysis in Christchurch. N Z Med J. 2000;113(1103):27-9. doi:doi:
32. Meier M, Utte A, Smith E, et al.. Survival and mortality analysis in two nephrology centers: A prospective observational study. Clin Nephrol. 2021;96(6):337-347. doi:doi:10.5414/cn110364
33. Menon MK, Naimark DM, Bargman JM, Vas SI, Oreopoulos DG. Long-term blood pressure control in a cohort of peritoneal dialysis patients and its association with residual renal function. Nephrol Dial Transplant. 2001;16(11):2207-13. doi:doi:10.1093/ndt/16.11.2207
34. Ploos van Amstel S, Noordzij M, Borzych-Duzalka D, et al.. Mortality in Children Treated With Maintenance Peritoneal Dialysis: Findings From the International Pediatric Peritoneal Dialysis Network Registry. Am J Kidney Dis. 2021;78(3):380-390. doi:doi:10.1053/j.ajkd.2020.11.031
35. Potter DE, San Luis E, Wipfler JE, Portale AA. Comparison of continuous ambulatory peritoneal dialysis and hemodialysis in children. Kidney Int Suppl. 1986;19:S11-4. doi:doi:
36. Prasad N, Gulati S, Gupta A, et al.. Continuous peritoneal dialysis in children: A single-centre experience in a developing country. Pediatric Nephrology. 2006;21(3):403-407. doi:doi:10.1007/s00467-005-2090-7
37. Rao CSS, Charan P, Naidu G, Swarnalatha G, Ram R, Dakshinamurty KV. A 2-year follow-up study of patients on automated peritoneal dialysis. Indian Journal of Nephrology. 2013;23(5):327-331. doi:doi:10.4103/0971-4065.116292
38. Sari F, Sarikaya M, Cetinkaya R, Gunes AJ, Eren M. Clinical Outcomes of Peritoneal Dialysis Patients at a Secondary State Hospital Nephrology Clinic. Turkish Nephrology Dialysis and Transplantation Journal. 2012;21(1):34-38. doi:doi:10.5262/tndt.2012.1001.06
39. Tsakiris DJ, Smith WG, Briggs JD, Junor BJ. Continuous ambulatory peritoneal dialysis: a three year experience. Scott Med J. 1986;31(2):79-84. doi:doi:10.1177/003693308603100204
40. Unsal A, Koc Y, Basturk T, et al.. Clinical outcomes and mortality in peritoneal dialysis patients: a 10-year retrospective analysis in a single center. Clin Nephrol. 2013;80(4):270-9. doi:doi:10.5414/cn107711
41. Ur-Rehman K, Housawi A, Al-Jifri A, Kielar M, Al-Ghamdi SM. Peritoneal dialysis for chronic kidney disease patients: a single-center experience in Saudi Arabia. Saudi J Kidney Dis Transpl. 2011;22(3):581-6. doi:doi:
42. van Stralen KJ, Borzych-Dualka D, Hataya H, et al.. Survival and clinical outcomes of children starting renal replacement therapy in the neonatal period. Kidney International. 2014;86(1):168-

174. doi:doi:10.1038/ki.2013.561

1. Verdalles U, Abad S, Aragoncillo I, et al.. Factors predicting mortality in elderly patients on dialysis. Nephron Clin Pract. 2010;115(1):c28-34. doi:doi:10.1159/000286347
2. Wang AY, Wang M, Lam CW, Chan IH, Lui SF, Sanderson JE. Heart failure in long-term peritoneal dialysis patients: a 4-year prospective analysis. Clin J Am Soc Nephrol. 2011;6(4):805-12. doi:doi:10.2215/cjn.07130810
3. Weaver DJ, Somers MJG, Martz K, Mitsnefes MM. Clinical outcomes and survival in pediatric patients initiating chronic dialysis: a report of the NAPRTCS registry. Pediatric Nephrology. 2017;32(12):2319-2330. doi:doi:10.1007/s00467-017-3759-4
4. Wei SS, Lee GS, Woo KT, Lim CH. Continuous ambulatory peritoneal dialysis in type II diabetics. Ann Acad Med Singap. 1993;22(4):629-33. doi:doi:
5. Wong PN, Mak SK, Lo KY, Tong GM, Wong Y, Wong AK. Adverse prognostic indicators in continuous ambulatory peritoneal dialysis patients without obvious vascular or nutritional comorbidities. Perit Dial Int. 2003;23:S109-15. doi:doi:
6. Xu H, Lindholm B, Lundström UH, et al.. Treatment practices and outcomes in incident peritoneal dialysis patients: the Swedish Renal Registry 2006-2015. Clin Kidney J. 2021;14(12):2539-2547. doi:doi:10.1093/ckj/sfab130
7. Yeoh LY, Sivaraman P. Factors that might adversely affect short-term survival of patients starting peritoneal dialysis and use of those factors to predict outcome--a single-center experience. Perit Dial Int. 2003;23:S116-20. doi:doi:
8. Zbroch E, Maciorkowska D, Malyszko J, Mysliwiec M. Influence of diabetes on hypotensive treatment in the population of dialyzed patients. Arterial Hypertension. 2012;16(2):85-92.
9. Zhang F, Liu H, Gong X, et al.. Risk factors for mortality in Chinese patients on continuous ambulatory peritoneal dialysis. Perit Dial Int. 2015;35(2):199-205. doi:doi:10.3747/pdi.2013.00164
10. Zhang Q, Ren H, Xie J, Li X, Huang X, Chen N. Causes of death in peritoneal dialysis patients with different kidney diseases and comorbidities: a retrospective clinical analysis in a Chinese center. Int Urol Nephrol. 2014;46(6):1201-7. doi:doi:10.1007/s11255-013-0561-5
11. Zhao C, Luo Q, Xia X, et al.. Risk score to predict mortality in continuous ambulatory peritoneal dialysis patients. Eur J Clin Invest. 2014;44(11):1095-103. doi:doi:10.1111/eci.12344

# Excluded: Irrelevant exposure and/or outcome (n = 46)

1. Abe M, Hamano T, Hoshino J, Wada A, Nakai S, Masakane I. Glycemic control and survival in peritoneal dialysis patients with diabetes: A 2-year nationwide cohort study. Sci Rep. 2019;9(1):3320. doi:doi:10.1038/s41598-019-39933-5
2. Ammirati AL, Dalboni MA, Cendoroglo M, et al.. The progression and impact of vascular calcification in peritoneal dialysis patients. Perit Dial Int. 2007;27(3):340-6. doi:doi:
3. Arogundade FA, Ishola DA, Sanusi AA, Akinsola A. An analysis of the effectiveness and benefits of peritoneal dialysis and haemodialysis using Nigerian made PD fluids. African journal of medicine and medical sciences. 2005;34(3):227‐233. doi:doi:
4. Ateş K, Ateş A, Kutlay S, Nergizoǧlu G, Karatan O. Total lymphocyte count in peripheral blood of peritoneal dialysis patients: Relationship to clinical parameters and outcome. Journal of Nephrology. 2004;17(2):246-252. doi:doi:
5. Ayazi K, Atabak S, Saghebi R, Ayazi S, Aryasepehr S. Evaluation of efficacy, survival rate and complications of peritoneal catheter placement of patients with end-stage renal disease. Saudi Med J. 2005;26(9):1391-3. doi:doi:
6. Bohorques R, Álvarez Y, Martínez A, Ballard Y, Pérez S, Gutiérrez F. Use of Home Peritoneal Dialysis by Cuba's Nephrology Institute, 2007-2012. MEDICC Rev. 2015;17(2):29-32. doi:doi:10.37757/mr2015.V17.N2.7
7. Borzych-Duzalka D, Bilginer Y, Ha IS, et al.. Management of anemia in children receiving chronic peritoneal dialysis. Journal of the American Society of Nephrology (JASN). 2013;24(4):665-676. doi:doi:10.1681/ASN.2012050433
8. Buffet A, Guillouët S, Lobbedez T, Ficheux M, Lanot A, Béchade C. Safety of peritoneal dialysis after nonrenal solid-organ transplantation. Peritoneal Dialysis International. 2018;38(1):37-43. doi:doi:10.3747/pdi.2017.00125
9. Cao L, Mou S, Fang W, et al.. Hyperleptinaemia, insulin resistance and survival in peritoneal dialysis patients. Nephrology. 2015;20(9):617-624. doi:doi:10.1111/nep.12491
10. Catalano C, Goodship TH, Tapson JS, et al.. Renal replacement treatment for diabetic patients in Newcastle upon Tyne and the Northern region, 1964-88. Bmj. 1990;301(6751):535-40. doi:doi:10.1136/bmj.301.6751.535
11. N, Sav NM, Karabel D, Yildirim A, Yildiz B. Serum albumin and von Willebrand factor: possible markers for early detection of vascular damage in children undergoing peritoneal dialysis. Clinical and Investigative Medicine. 2016;39(4):E111-E119. doi:doi:
12. Choi SR, Lee SC, Kim BS, et al.. Comparative study of renal replacement therapy in Korean diabetic end-stage renal disease patients: a single center study. Yonsei Medical Journal. 2003;44(3):454-462. doi:doi:10.3349/ymj.2003.44.3.454
13. Civilibal M, Caliskan S, Oflaz H, et al.. Traditional and "new" cardiovascular risk markers and factors in pediatric dialysis patients. Pediatr Nephrol. 2007;22(7):1021-9. doi:doi:10.1007/s00467-007-0451-0
14. Cnossen TT, Usvyat L, Kotanko P, et al.. Comparison of outcomes on continuous ambulatory peritoneal dialysis versus automated peritoneal dialysis: results from a USA database. Perit Dial Int. 2011;31(6):679-84. doi:doi:10.3747/pdi.2010.00004
15. Cornelis T, Rioux JP, Bargman JM, Chan CT. Home dialysis is a successful strategy in nonrenal solid organ transplant recipients with end-stage renal disease. Nephrol Dial Transplant. 2010;25(10):3425-9. doi:doi:10.1093/ndt/gfq373
16. Dong J, Li Y, Yang Z, Luo J, Zuo L. Time-dependent associations between total sodium removal and mortality in patients on peritoneal dialysis. Perit Dial Int. 2011;31(4):412-21. doi:doi:10.3747/pdi.2010.00103
17. Feng X, Wen Y, Peng FF, Wang N, Zhan X, Wu X. Association between aminotransferase/alanine aminotransferase ratio and cardiovascular disease mortality in patients on peritoneal dialysis: a multi-center retrospective study. BMC Nephrol. 2020;21(1):209. doi:doi:10.1186/s12882-020-01840-7
18. Feng X, Zhan X, Wen Y, et al.. Hyperlipidemia and mortality in patients on peritoneal dialysis. BMC Nephrol. 2022;23(1):342. doi:doi:10.1186/s12882-022-02970-w
19. Fernandes N, Bastos MG, Cassi HV, et al.. The Brazilian Peritoneal Dialysis Multicenter Study (BRAZPD) : characterization of the cohort. Kidney Int Suppl. 2008;(108):S145-51. doi:doi:10.1038/sj.ki.5002616
20. Foley RN, Parfrey PS, Harnett JD, Kent GM, Murray DC, Barre PE. The impact of anemia on cardiomyopathy, morbidity, and mortality in end-stage renal disease. Am J Kidney Dis. 1996;28(1):53-61. doi:doi:10.1016/s0272-6386(96)90130-4
21. Gheissari A, Sirous M, Hajzargarbashi T, Kelishadi R, Merrikhi A, Azhir A. Carotid intima- media thickness in children with end-stage renal disease on dialysis. Indian J Nephrol. 2010;20(1):29-33. doi:doi:10.4103/0971-4065.62095
22. Gu W, Yi C, Yu X, Yang X. Metabolic Syndrome and Mortality in Continuous Ambulatory Peritoneal Dialysis Patients: A 5-Year Prospective Cohort Study. Kidney and Blood Pressure Research. 2019;44(5):1026-1035. doi:doi:10.1159/000502145
23. Hendawy A, Pouteil-Noble C, Villar E, Boissonnat P, Sebbag L. Chronic renal failure and end- stage renal disease are associated with a high rate of mortality after heart transplantation. Transplant Proc. 2005;37(2):1352-4. doi:doi:10.1016/j.transproceed.2004.12.276
24. Karadağ S, Gürsu M, Öztürk S, et al.. Periton Diyalizi Hastalarında Yeni Bir Endotel Fonksiyonu Belirteci: Apelin. Medical Bulletin of Haseki / Haseki Tip Bulteni. 2013;51(1):5-10. doi:doi:10.4274/Haseki.808
25. Karthikeyan B, Sharma RK, Kaul A, Gupta A, Prasad N, Bhadauria DS. Clinical characteristics, patient and technique survival in elderly patients on peritoneal dialysis. Indian Journal of Nephrology. 2019;29(5):334-339. doi:doi:10.4103/ijn.IJN_377_18
26. Laakkonen H, Hölttä T, Lönnqvist T, Holmberg C, Rönnholm K. Peritoneal dialysis in children under two years of age. Nephrol Dial Transplant. 2008;23(5):1747-53. doi:doi:10.1093/ndt/gfn035
27. Lee CC, Sun CY, Wu MS. Long-term modality-related mortally analysis in incident dialysis Patients. Peritoneal Dialysis International. 2009;29(2):182-190. doi:doi:10.1177/089686080902900213
28. Liu X, Guo Q, Feng X, et al.. Alkaline phosphatase and mortality in patients on peritoneal dialysis. Clinical Journal of the American Society of Nephrology. 2014;9(4):771-778. doi:doi:10.2215/CJN.08280813
29. Lynn KL, McGregor DO, Moesbergen T, Buttimore AL, Inkster JA, Wells JE. Hypertension as a determinant of survival for patients treated with home dialysis. Kidney Int. 2002;62(6):2281-7. doi:doi:10.1046/j.1523-1755.2002.00685.x
30. Mailloux LU, Kapikian N, Napolitano B, et al.. Home hemodialysis: Patient outcomes during a 24-year period of time from 1970 through 1993. Advances in Renal Replacement Therapy. 1996;3(2):112-119. doi:doi:10.1016/S1073-4449(96)80050-1
31. Mimura T, Takenaka T, Kanno Y, Aoki H, Ohshima J, Suzuki H. Comparison of changes in pulse wave velocity in patients on continuous ambulatory peritoneal dialysis and hemodialysis one year after introduction of dialysis therapy. Adv Perit Dial. 2005;21:139-45. doi:doi:
32. Mitsnefes MM, Daniels SR, Schwartz SM, Khoury P, Strife CF. Changes in left ventricular mass in children and adolescents during chronic dialysis. Pediatr Nephrol. 2001;16(4):318-23. doi:doi:10.1007/s004670000557
33. Mitsnefes MM, Daniels SR, Schwartz SM, Meyer RA, Khoury P, Strife CF. Severe left ventricular hypertrophy in pediatric dialysis: prevalence and predictors. Pediatr Nephrol. 2000;14(10):898-902. doi:doi:10.1007/s004670000303
34. Munib S. Continuous ambulatory peritoneal dialysis in Khyber Pukhtonkhuwa province of Pakistan and adjoining areas of Afghanistan. Rawal Medical Journal. 2012;37(3):281-285. doi:doi:
35. Nakai S, Shinzato T, Nagura Y, et al.. An overview of regular dialysis treatment in Japan as of 31 December 2003. Therapeutic Apheresis and Dialysis. 2005;9(6):431-458. doi:doi:10.1111/j.1744-9987.2005.00328.x
36. Refaat H, Sany D, Mohab A, Ezzat H. Comparing Dialysis Modality and Cardiovascular Mortality in Patients on Hemodialysis and Peritoneal Dialysis. Adv Perit Dial. 2016;32:22-31. doi:doi:
37. Rottembourg J, Issad B, Allouache M, Baumelou A, Deray G, Jacobs C. Clinical aspects of continuous ambulatory and continuous cyclic peritoneal dialysis in diabetic patients. Perit Dial Int. 1989;9(4):289-94. doi:doi:
38. Sipahioglu MH, Kucuk H, Unal A, et al.. Impact of arterial stiffness on adverse cardiovascular outcomes and mortality in peritoneal dialysis patients. Perit Dial Int. 2012;32(1):73-80. doi:doi:10.3747/pdi.2010.00186
39. Szeto CC, Kwan BC, Chow KM, Leung CB, Law MC, Li PK. Prognostic value of arterial pulse wave velocity in peritoneal dialysis patients. Am J Nephrol. 2012;35(2):127-33. doi:doi:10.1159/000335580
40. Tian SL, Murphy M, Han QF, Lu XH, Wang T. Prevalence and risk factors for peripheral artery disease among patients on maintenance peritoneal dialysis. Blood Purif. 2010;30(1):50-5. doi:doi:10.1159/000317121
41. Tong M, Wang Y, Ni J, et al.. Clinical features of patients treated by peritoneal dialysis for over a decade. Am J Clin Exp Urol. 2017;5(3):49-54. doi:doi:
42. Wang AY, Wang M, Woo J, et al.. A novel association between residual renal function and left ventricular hypertrophy in peritoneal dialysis patients. Kidney Int. 2002;62(2):639-47. doi:doi:10.1046/j.1523-1755.2002.00471.x
43. Weber J, Mettang T, Mayer-Wehrstein R, Kuhlmann U. Continuous ambulatory peritoneal dialysis: Ten years' patient and method survival rate, incidence of peritonitis, and dialysis effectiveness. Deutsche Medizinische Wochenschrift. 1991;116(17):641-648. doi:doi:10.1055/s- 2008-1063660
44. Wu V, Huang J, Wu M, et al.. The effect of iron stores on corrected QT dispersion in patients undergoing peritoneal dialysis. American Journal of Kidney Diseases. 2004;44(4):720-728. doi:doi:
45. Xu T, Xie J, Zong X, Wang W, Ren H, Chen N. Pulse Wave Velocity: A Valuable Predictor for Cardio-Cerebrovascular Disease and Death in PD Patients. Blood Purif. 2015;40(3):203-8. doi:doi:10.1159/000433516
46. Yong K, Dogra G, Boudville N, Lim W. Increased Inflammatory Response in Association with the Initiation of Hemodialysis Compared with Peritoneal Dialysis in a Prospective Study of End- Stage Kidney Disease Patients. Perit Dial Int. 2018;38(1):18-23. doi:doi:10.3747/pdi.2017.00060

# Excluded: Combined mortality of peritoneal dialysis and hemodialysis patients (n= 16)

1. Aniort J, Kaysi S, Garrouste C, et al.. CKD complications in kidney-transplanted patients going back to dialysis: impact on patients outcomes. Journal of Nephrology. 2018;31(1):147-155. doi:doi:10.1007/s40620-017-0449-z
2. Arogundade FA, Sanusi AA, Hassan MO, Akinsola A. The pattern, clinical characteristics and outcome of ESRD in Ile-Ife, Nigeria: is there a change in trend? Afr Health Sci. 2011;11(4):594- 601. doi:doi:
3. Ayer A, Mills C, Donovan C, Christenson RH, Ganz P, Dubin RF. Associations of microvascular dysfunction with cardiovascular outcomes: The cardiac, endothelial function and arterial stiffness in ESRD (CERES) cohort. Hemodial Int. 2019;23(1):58-68. doi:doi:10.1111/hdi.12675
4. Bakkaloglu SA, Kandur Y, Serdaroglu E, et al.. Time-averaged hemoglobin values, not hemoglobin cycling, have an impact on outcomes in pediatric dialysis patients. Pediatric Nephrology. 2018;33(11):2143-2150. doi:doi:10.1007/s00467-018-4013-4
5. Cafka M, Rroji M, Seferi S, et al.. Inflammation, Left Ventricular Hypertrophy, and Mortality in End-stage Renal Disease. Iran J Kidney Dis. 2016;10(4):217-23. doi:doi:
6. Cancarini GC, Sandrini M, Vizzardi V, et al.. Long-term peritoneal dialysis outcome in a single center. Peritoneal Dialysis International. 2000;20:S121-S126. doi:doi:10.1177/089686080002002s24
7. De Lima JJ, da Fonseca JA, Godoy AD. Baseline variables associated with early death and extended survival on dialysis. Ren Fail. 1998;20(4):581-7. doi:doi:10.3109/08860229809045150
8. Duranti E, Imperiali P, Sasdelli M. Is hypertension a mortality risk factor in dialysis? Kidney Int Suppl. 1996;55:S173-4. doi:doi:
9. Enríquez J, Bastidas M, Mosquera M, et al.. Survival on chronic dialysis: 10 years' experience of a single Colombian center. Adv Perit Dial. 2005;21:164-7. doi:doi:
10. Herrera L, Gil F, Sanabria M. Hemodialysis vs Peritoneal Dialysis: Comparison of Net Survival in Incident Patients on Chronic Dialysis in Colombia. Canadian Journal of Kidney Health and Disease. 2021;8doi:doi:10.1177/2054358120987055
11. Hosseini M, Roshani S, Ataei N, et al.. Prevalence and Risk Factors of Technique Failure in Peritoneal Dialysis of Iranian Children and Adolescents. International Journal of Pediatrics- Mashhad. 2019;7(11):10377-10385. doi:doi:10.22038/ijp.2019.44136.3660
12. Katafuchi E, Nakayama M, Tanaka S, et al.. Comparison of prognostic values of daytime and night-time systolic blood pressures on renal outcomes in patients with Chronic kidney disease. Circulation Journal. 2017;81(10):1454-1462. doi:doi:10.1253/circj.CJ-17-0063
13. Lunde NM, Port FK, Wolfe RA, Guire KE. Comparison of mortality risk by choice of CAPD versus hemodialysis among elderly patients. Adv Perit Dial. 1991;7:68-72. doi:doi:
14. Madziarska K, Weyde W, Krajewska M, et al.. Elderly dialysis patients: analysis of factors affecting long-term survival in 4-year prospective observation. International Urology and Nephrology. 2012;44(3):955-961. doi:doi:10.1007/s11255-012-0166-4
15. Plantinga LC, Fink NE, Levin NW, et al.. Early, intermediate, and long-term risk factors for mortality in incident dialysis patients: The choices for healthy outcomes in caring for ESRD (CHOICE) study. American Journal of Kidney Diseases. 2007;49(6):831-840. doi:doi:10.1053/j.ajkd.2007.03.017
16. Suzuki T, Kanno Y, Nakamoto H, Okada H, Sugahara S, Suzuki H. Peritoneal dialysis versus hemodialysis: a five-year comparison of survival and effects on the cardiovascular system, erythropoiesis, and calcium metabolism. Adv Perit Dial. 2003;19:148-54. doi:doi:

# Excluded: Only subtype mortality reported (n = 4)

1. Choi SR, Lee YK, Cho Park H, et al.. Clinical significance of central systolic blood pressure in LV diastolic dysfunction and CV mortality. PLoS One. 2021;16(5):e0250653. doi:doi:10.1371/journal.pone.0250653
2. Li W, Xu R, Wang Y, et al.. Association of body mass index and uncontrolled blood pressure with cardiovascular mortality in peritoneal dialysis patients. J Hum Hypertens. 2019;33(2):106-

114. doi:doi:10.1038/s41371-018-0107-5

1. Wang Z, Yu D, Cai Y, Zhao B, Zhang X, Zhao Z. Optimal cut-off threshold in pulse pressure predicting cardiovascular death among newly diagnosed end-stage renal disease patients: A prospective cohort study. Medicine (Baltimore). 2019;98(27):e16340. doi:doi:10.1097/md.0000000000016340
2. Zhang X, Yu D, Cai Y, et al.. Dose-Response Between Cardiovascular Risk Factors and Cardiovascular Mortality Among Incident Peritoneal Dialysis Patients. Kidney Blood Press Res. 2018;43(2):628-638. doi:doi:10.1159/000489289

# Excluded: Insufficient quantitative data (n = 8)

1. Abraham G, Kumar V, Nayak KS, et al.. Predictors of long-term survival on peritoneal dialysis in South India: a multicenter study. Perit Dial Int. 2010;30(1):29-34. doi:doi:10.3747/pdi.2008.00028
2. Gorsane I, Hamida SB, Hamida FB, Ounissi M, Harzallah A, Abdallah TB. Peritoneal dialysis in diabetis patients. Tunisie Medicale. 2019;97(8):1017-1023. doi:doi:
3. Hakemi MS, Golbabaei M, Nassiri A, et al.. Predictors of patient survival in continuous ambulatory peritoneal dialysis: 10-year experience in 2 major centers in Tehran. Iran J Kidney Dis. 2010;4(1):44-9. doi:doi:
4. Rottembourg J, Issad B, Allouache M, et al.. [Clinical aspects of continuous ambulatory peritoneal dialysis in diabetics]. Nephrologie. 1988;9(5):227-32. doi:doi:
5. Wong TY, Szeto CC, Chow KM, Chan JC, Li PK. Prognostic role of serum ACE activity on outcome of type 2 diabetic patients on chronic ambulatory peritoneal dialysis. Am J Kidney Dis. 2002;39(5):1054-60. doi:doi:10.1053/ajkd.2002.32789
6. Wu C-F, Lee Y-F, Lee W-J, et al.. Severe aortic arch calcification predicts mortality in patients undergoing peritoneal dialysis. Journal of the Formosan Medical Association. 2017;116(5):366-

372. doi:doi:10.1016/j.jfma.2016.06.006

1. Yang Y, Yang Y, Liu YH, Ye Y. [Effect of peritoneal dialysis in elderly patients]. Hunan Yi Ke Da Xue Xue Bao. 2000;25(2):161-2. doi:doi:
2. Zimmerman SW, Johnson CA, O'Brien M. Survival of diabetic patients on continuous ambulatory peritoneal dialysis for over five years. Peritoneal Dialysis Bulletin. 1987;7(1):26-29. doi:doi:10.1177/0896860887007001
